# A Mathematical Model of Lymphangiogenesis in a Zebrafish Embryo

**DOI:** 10.1007/s11538-017-0248-7

**Published:** 2017-02-23

**Authors:** Kenneth Y. Wertheim, Tiina Roose

**Affiliations:** Faculty of Engineering and the Environment, University of Southampton, Highfield Campus, Southampton, SO17 1BJ UK

**Keywords:** Mathematical model, Lymphangiogenesis, Zebrafish, VEGFC, Collagen I, MMP2

## Abstract

The lymphatic system of a vertebrate is important in health and diseases. We propose a novel mathematical model to elucidate the lymphangiogenic processes in zebrafish embryos. Specifically, we are interested in the period when lymphatic endothelial cells (LECs) exit the posterior cardinal vein and migrate to the horizontal myoseptum of a zebrafish embryo. We wonder whether vascular endothelial growth factor C (VEGFC) is a morphogen and a chemotactic factor for these LECs. The model considers the interstitial flow driving convection, the reactive transport of VEGFC, and the changing dynamics of the extracellular matrix in the embryo. Simulations of the model illustrate that VEGFC behaves very differently in diffusion and convection-dominant scenarios. In the former case, it must bind to the matrix to establish a functional morphogen gradient. In the latter case, the opposite is true and the pressure field is the key determinant of what VEGFC may do to the LECs. Degradation of collagen I, a matrix component, by matrix metallopeptidase 2 controls the spatiotemporal dynamics of VEGFC. It controls whether diffusion or convection is dominant in the embryo; it can create channels of abundant VEGFC and scarce collagen I to facilitate lymphangiogenesis; when collagen I is insufficient, VEGFC cannot influence the LECs at all. We predict that VEGFC is a morphogen for the migrating LECs, but it is not a chemotactic factor for them.

## Introduction

The lymphatic system of a vertebrate plays many roles in health and in diseases. Most importantly, it drains the interstitial fluid of its tissues back to the blood vasculature, thereby maintaining tissue homoeostasis and absorbing intestinal lipids (Margaris and Black 2012; Schulte-Merker et al. 2011). Furthermore, various immune cells reside in the lymph nodes distributed throughout the lymphatic system; they filter the circulating lymph (Margaris and Black 2012). If the lymphatic system malfunctions, a medical condition called lymphoedema ensues; it is characterised by swelling and pain due to a build-up of interstitial fluid (Margaris and Black 2012).

In a vertebrate, lymphatic vessels are present in most organs except avascular tissues like cartilage (Schulte-Merker et al. 2011; Louveau et al. 2015). Like Roose and Tabor (2013), we will classify them into primary and secondary lymphatics. Margaris and Black (2012) is a detailed review of both categories. The primary lymphatics, also called initial lymphatics, are the entry points of a lymphatic system. They are lined by a monolayer of nonfenestrated lymphatic endothelial cells (LECs). They drain their surrounding tissues of excessive fluid passively, a process driven by fluctuations in their interstitial pressures. The resulting lymph is delivered into the larger secondary lymphatics. Also known as collecting ducts, they have walls that contain smooth muscle cells to propel lymphatic circulation by contractions; the muscles, arteries, and organs nearby also add to the propelling forces. The secondary lymphatics drain into various veins, thereby returning lymph to the vertebrate’s blood vasculature.

**Fig. 1:**
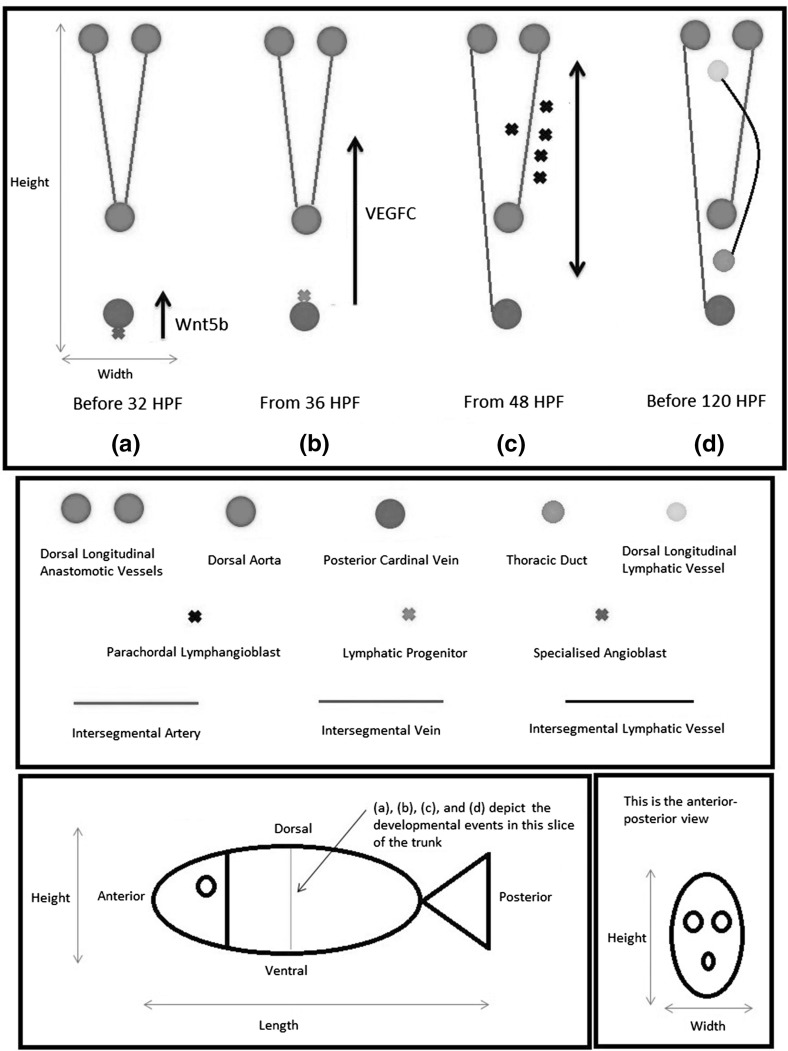
(Color figure online) Developmental steps that generate the lymphatic system in the trunk of a zebrafish embryo. *a*–*d* A slice of the trunk cut in the ventral–dorsal direction, so they depict the developmental events in the anterior–posterior view. This particular slice of the trunk has a pair of intersegmental arteries (aISVs) and a pair of lymphatic sprouts, one of which fuses with an aISV to from an intersegmental vein (vISV). There are 30 slices like this one in the trunk. When the parachordal lymphangioblasts (PLs) reach where the thoracic duct and the dorsal longitudinal lymphatic vessel lie in the ventral–dorsal slice depicted, they migrate anteriorly and posteriorly to connect with the PLs from the remaining 29 slices

How such an important and complex structure develops is incompletely understood. In this paper, we will investigate lymphangiogenesis in zebrafish (*Danio rerio*) embryos. Lymphangiogenesis is the development and proliferation of new lymphatics by sprouting from veins and/or any pre-existing lymphatic structures (Ji 2006). Zebrafish is a model organism widely used for studying vascular development (Gore et al. 2012). According to Florence Sabin’s conceptual model (Sabin 1902), the lymphatic vasculature of a vertebrate stems from the blood vasculature. This mechanism is generally accepted by the scientific community nowadays (Schulte-Merker et al. 2011). In zebrafish, various venous origins contribute the precursor cells which will form the trunk lymphatics, the facial lymphatics, the lateral lymphatics, and the intestinal lymphatics (Koltowska et al. 2013). Our focus is on the trunk lymphatics. The developmental steps which generate the lymphatic vasculature in a zebrafish trunk are illustrated in Fig. 1 and described as follows.

Within 24 h post-fertilisation (HPF), Wnt5b secreted by the endoderm commits the cells in the ventral wall of the posterior cardinal vein (PCV) to the lymphatic fate; the resulting LECs will translocate to the dorsal side of the PCV by 30 HPF (Nicenboim et al. 2015). At 32 HPF, most of the blood vasculature is fully formed, including the dorsal aorta (DA), the PCV, a set of intersegmental arteries (aISVs), and a pair of dorsal longitudinal anastomotic vessels (DLAVs) (Koltowska et al. 2013).

At around 36 HPF, 30 pairs of secondary sprouts emerge from the dorsal side of the PCV and migrate dorsally (van Impel and Schulte-Merker 2014). The LECs constituting these sprouts only exit the PCV when they are stimulated by the growth factor VEGFC (Hogan et al. 2009). In mice at least, only the LECs that have exited the veins can express podoplanin (Koltowska et al. 2013). Although podoplanin is absent in zebrafish (Chen et al. 2014), the genetic programmes regulating lymphangiogenesis in zebrafish and mice are more similar than different, as argued in van Impel and Schulte-Merker (2014). This leads us to assume that the PCV-derived LECs will change their gene expression profile after exiting the PCV.

At approximately 48 HPF, half of the secondary sprouts are already fused with their adjacent aISVs to form a set of intersegmental veins (vISVs); the remaining sprouts aggregate in a region named horizontal myoseptum, forming a pool of parachordal lymphangioblasts (PLs) (van Impel and Schulte-Merker 2014). The horizontal myoseptum expresses the ligand Cxcl12a which binds to the receptor Cxcr4 expressed by the LECs constituting the sprouts, thus ensuring the dorsally migrating LECs turn laterally when they reach the horizontal myoseptum (Cha et al. 2012). Further guidance cues for the LECs are thought to be provided by the motor neuron axons positioned along the horizontal myoseptum (Cha et al. 2012). After the LECs form the pool of PLs, they continue to express Cxcr4; they will migrate both ventrally and dorsally along their adjacent aISVs which express the ligand Cxcl12b (Cha et al. 2012).

Before 120 HPF, the PLs form the thoracic duct (TD) between the DA and the PCV, as well as the dorsal longitudinal lymphatic vessel (DLLV) below the DLAVs (van Impel and Schulte-Merker 2014). These two lymphatic vessels are connected via a set of intersegmental lymphatic vessels (ISLVs) which are close to the aISVs (van Impel and Schulte-Merker 2014). At this stage, the PCV expresses Cxcl12a and the DA expresses Cxcl12b, thus ensuring the ventrally migrating PLs will end up between the two blood vessels (Cha et al. 2012). Once they reach where the DLLV and TD should lie in a ventral–dorsal slice, the PLs will migrate anteriorly and posteriorly to connect with the PLs from the other ventral–dorsal slices in the trunk, thus ensuring the two lymphatic vessels are continuous.

There are several missing details in this developmental process. The LECs exit the PCV under the influence of VEGFC. During their dorsal migration, their gene expression profile probably changes, similar to their counterparts in a mouse embryo. Although we know that Cxcl12a causes the LECs to aggregate along the horizontal myoseptum, we do not know what causes them to migrate dorsally instead of ventrally or laterally from the PCV. Neither do we know what changes their gene expression profile during migration. In short, we are uncertain about what happens between (b) and (c) in Fig. 1.

We know that VEGFC promotes survival, proliferation, and migration in LECs through the PI3K/AKT and RAS/RAF/ERK signalling pathways; the PI3K/AKT pathway regulates their migration, while the RAS/RAF/ERK pathway controls lymphatic fate specification (Mäkinen et al. 2001; Deng et al. 2013). A possibility is that VEGFC is more than a growth factor for the PCV-derived LECs. It may be a chemotactic factor and a morphogen too. By chemotactic factor, we mean a chemical which directs the migrating LECs dorsally to the horizontal myoseptum. By morphogen, we mean a chemical which provides positional information to the LECs so that they alter their gene expression after exiting the PCV. In Sect. 2, we will build a mathematical model of the spatiotemporal dynamics of VEGFC in the trunk of a zebrafish embryo. In Sect. 3, we will solve the model numerically under different conditions to explore the aforementioned possibilities. In Sect. 4, we will integrate the simulation results into answers to our research questions about lymphangiogenesis.

## Development of the Mathematical Model

In the following subsection, we will represent a zebrafish’s trunk with a simplified geometry. Then, we will use Brinkman’s equation to model the interstitial flow in the trunk. After that, we will use different forms of the reaction–diffusion–convection equation to model the reactive transport of VEGFC in the interstitial space of the trunk, as well as the changing composition of the interstitial space itself. We will complete the model by connecting the composition of the interstitial space to the interstitial flow. The resulting mathematical model will be a single framework which integrates these biochemical and biophysical phenomena. Then, we will parametrise, nondimensionalise, and simplify this mathematical model.

### Geometry

According to van Impel and Schulte-Merker (2014), the blood and lymphatic vasculatures in a zebrafish trunk are spatially periodic in the anterior–posterior direction as defined in Fig. 1. Exceptions are the three sets of intersegmental vessels: the aISVs, the vISVs, and the ISLVs, which appear at certain points on the anterior–posterior axis only; they extend in the ventral–dorsal direction as defined in Fig. 1. The secondary sprouts emerge next to the aISVs, so the three sets of intersegmental vessels coalign in 30 ventral–dorsal slices of the trunk (Isogai et al. 2003). Two adjacent slices are about 75 $\upmu \hbox {m}$ apart (Coffindaffer-Wilson et al. 2011b), so they can be considered independently. Each slice is similar to the one shown in Fig. 1. We will take advantage of these features and model one slice only.

However, we will not model the aISVs because our interest is from 36 to 48 HPF, the period when the PCV-derived lymphatic progenitors migrate to the horizontal myoseptum and differentiate *en route*. These events are not dependent on the aISVs (Bussmann et al. 2010).

There are a pair of DLAVs, but the distance between them is small. Representing them as two separate tubes requires a high-resolution grid, so we will model one DLAV only and double the flux into this vessel.

**Fig. 2:**
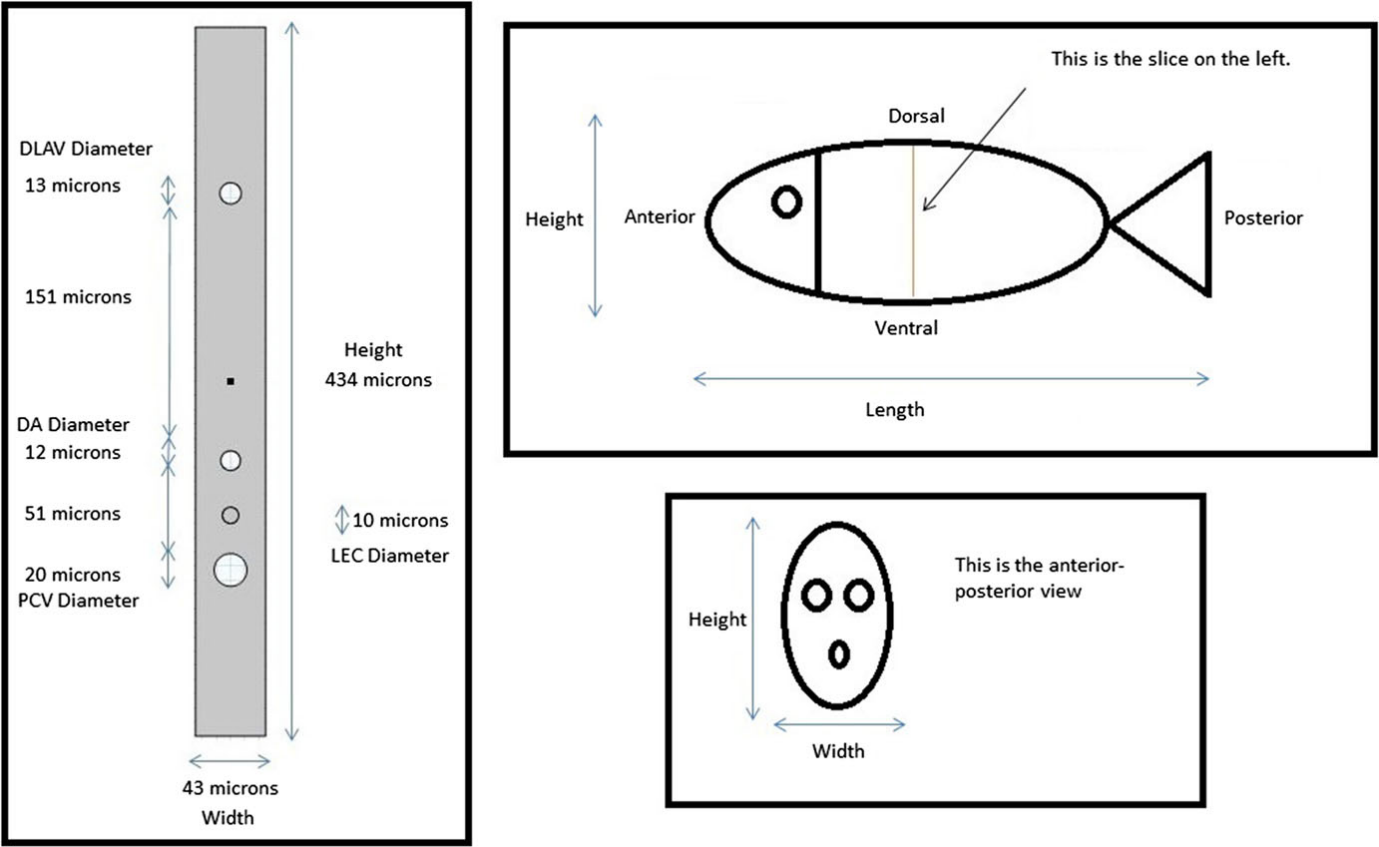
Idealised geometry of a ventral–dorsal slice of a zebrafish trunk between 36 and 48 h post-fertilisation. This figure shows the idealised geometry in the anterior–posterior view. This slice is one of the 30 slices with secondary sprouts from the posterior cardinal vein. The *empty circles* are, from *top* to *bottom*, the dorsal longitudinal anastomotic vessel (DLAV), the dorsal aorta (DA), and the posterior cardinal vein (PCV). The *solid circle* is a lymphatic endothelial cell (LEC) which has exited the posterior cardinal vein; it is halfway between the dorsal aorta and the posterior cardinal vein. The *dot in the middle* of the figure indicates the horizontal myoseptum, which is the destination of the LEC. In this study, we consider the LEC to be stationary

Based on the above assumptions, we can build an idealised geometry of the trunk between 36 and 48 HPF. The geometry is shown in Fig. 2. The LEC is located halfway between the DA and the PCV. It represents an LEC on its way to the horizontal myoseptum, but it is stationary in our model. For the purpose of model development, we will divide the geometry into two domains: the LEC and the interstitial space, which is the whole geometry minus the LEC. The dimensions of the geometry and its internal structures are summarised in Table 1.

**Table 1:** Dimensions of the idealised geometry and its internal structures

Quantity measured	Time	Measurement ($\upmu \hbox {m}$)	References
Total height	96 HPF	434	McGee et al. (2012)
Total width	72 HPF	43	Hermans et al. (2010)
PCV diameter	96 HPF	20	Coffindaffer-Wilson et al. (2011b)
DA diameter	96 HPF	12	Coffindaffer-Wilson et al. (2011b)
DLAV diameter	96 HPF	13	Coffindaffer-Wilson et al. (2011b)
PCV-DA distance	96 HPF	51	Coffindaffer-Wilson et al. (2011b)
DA-DLAV distance	96 HPF	151	Coffindaffer-Wilson et al. (2011b)
LEC diameter	–	10	Yaniv et al. (2006)

*PCV* posterior cardinal vein, *DA* dorsal aorta, *DLAV* dorsal longitudinal anastomotic vessel, *LEC* lymphatic endothelial cell, *HPF* hours post-fertilisation

### Interstitial Flow

Next, we will consider the interstitial flow in the trunk. It is driven by the pressure differences between the zebrafish’s blood vasculature, interstitial space, and lymphatic vasculature (Swartz and Fleury 2007). Clearly, our representation of the zebrafish trunk does not include a lymphatic vasculature. However, the blood circulation in a zebrafish begins by 30 HPF (Iida et al. 2010), so there is already an interstitial flow at the beginning of our time frame of interest. We need to incorporate this physical phenomenon into our mathematical model. On the other hand, we will not consider the flow’s effects on the LEC in our model trunk. In general, the shear stresses from a flow can induce intracellular and functional changes in cells (Shi and Tarbell 2011; Ng et al. 2004). However, the intracellular details necessary for the calculation of its mechanical responses are beyond the scope of this tissue-level model.

Our mathematical model of the interstitial flow relies on several assumptions. First, as in Coffindaffer-Wilson et al. (2011a), we will assume that the DA has the highest blood pressure in a zebrafish. The images in Coffindaffer-Wilson et al. (2011b) show that the DA, PCV, and DLAV have diameters comparable to a single cell. It is therefore reasonable to treat them as leaky capillaries (Jain 1987). The high DA pressure will force blood plasma into the interstitial space by paracellular transport, making the DA the inlet for fluid flow in our model. Second, we will assume a constant density for the resulting interstitial fluid. Third, we will only model the interstitial flow in the interstitial space because the LEC is separated by its cell membrane. Fourth, we will assume there are no sources or sinks of fluid in the interstitial space. Fifth, we will use a constant permeability for all three blood vessels because they are all assumed to behave like one-cell-thick capillaries. Sixth, we will ignore the pulsating nature of blood flow in this mathematical model. Finally, we will assume that the interstitial flow is at a steady state.

The interstitial space consists of the aforementioned interstitial fluid and an extracellular matrix (ECM), the latter of which is a porous medium. Therefore, the interstitial flow can be described by Darcy’s law. However, Darcy’s law does not permit the use of no-slip boundary conditions on the surfaces of internal structures, such as the blood vessels and the LEC in our geometry. More significantly, Darcy’s law assumes a homogeneous medium. In the next subsection, we will expand the model to include the remodelling events which degrade the ECM wherein channels may form. Darcy’s law cannot model these regions accurately. Brinkman’s equation can overcome both limitations. Using *P* (mmHg) to represent the pressure field in the interstitial space, $\mu$ (cP) the dynamic viscosity of the interstitial fluid, $\kappa$ ($\hbox {cm}^{2}$) the specific hydraulic conductivity of the ECM, and $\varvec{u}$ (µm/s) the interstitial fluid velocity, we can write Brinkman’s equation as

1 $$\begin{aligned} \varvec{\nabla }{P}=-\frac{\mu }{\kappa }\varvec{u}+\mu \varvec{\nabla }^2\varvec{u}. \end{aligned}$$

There are two dependent variables in Eq. (1): *P* and $\varvec{u}$, so we need another equation to define the flow problem. Because the interstitial fluid has a constant density and there are no sources or sinks of it in the interstitial space, conservation of mass is given by

2 $$\begin{aligned} \varvec{\nabla } \cdot {\varvec{u}}=0. \end{aligned}$$

To solve the flow problem, we need some boundary conditions. The fluxes out of the DA and into the PCV and the DLAV can be modelled by an equation describing the permeability of a vessel (Jain 1987). It is a linear relation between a transvascular flux and the transvascular pressure drop driving it. We will define $\varvec{x}$ as the position vector in our geometry and $\varvec{n}$ as a normal vector pointing out of the domain it resides in. Because the three normal vectors on the blood vessels point out of the interstitial space, they point into the vessels. Our definition also means a mass flux into the interstitial space is positive.

We will use $\rho$ ($\hbox {kg/m}^3$) to represent the density of the interstitial fluid, $L_\mathrm{DA}$ (cm/Pa/s) the DA vascular permeability, and $P^\mathrm{DA}$ (mmHg) the pressure inside the DA. Mathematically, the relation gives the mass flux from the DA surface ($\partial \varOmega _\mathrm{DA}$) into the interstitial space as

3 $$\begin{aligned} -\varvec{n} \cdot {(\rho \varvec{u})} = \rho L_\mathrm{DA} (P^\mathrm{DA}-P) \qquad \varvec{x} \in \partial \varOmega _\mathrm{DA}. \end{aligned}$$

We can derive the boundary conditions on the PCV and DLAV surfaces ($\partial \varOmega _\mathrm{PCV}$ and $\partial \varOmega _\mathrm{DLAV}$) along the same line to give

4 $$\begin{aligned} -\varvec{n} \cdot {\left( \rho \varvec{u}\right) }= & {} L_\mathrm{PCV} \left( P^\mathrm{PCV}-P\right) \qquad \varvec{x} \in \partial \varOmega _\mathrm{PCV} \; \text {and} \end{aligned}$$

5 $$\begin{aligned} -\varvec{n} \cdot {\left( \rho \varvec{u}\right) }= & {} 2 L_\mathrm{DLAV} \left( P^\mathrm{DLAV}-P\right) \qquad \varvec{x} \in \partial \varOmega _\mathrm{DLAV}. \end{aligned}$$

The multiplicative factor of 2 in Eq. (5) is there because we are representing two paired DLAVs as one vessel.

Finally, we will impose no-slip boundary conditions on the four outer boundaries of the geometry, collectively labelled $\partial \varOmega _{x,y}$, and the LEC surface, seen from the interstitial space domain, $\partial \varOmega _\mathrm{LEC/IS+}$. They are represented by

6 $$\begin{aligned} \varvec{u} = 0 \qquad \varvec{x} \in \partial \varOmega _{x,y} \; \text {and} \; \partial \varOmega _\mathrm{LEC/IS+}. \end{aligned}$$

### Reactive Transport of VEGFC and Extracellular Matrix Remodelling

In this subsection, we will add a biochemical reaction network to the mathematical model. We will model the transport phenomena of the participating biochemical species too.

VEGFC is synthesised as a preproprotein called proVEGFC; it has an N-terminal signal sequence followed by an N-terminal propeptide, then the VEGF homology, and finally a cysteine-rich C-terminal segment (Joukov et al. 1996, 1997; Siegfried et al. 2003). proVEGFC undergoes cleavage intracellularly and extracellularly (Joukov et al. 1997). After intracellular processing, proVEGFC will become a tetramer which has a molecular weight of 120 kDa and it will be secreted (Joukov et al. 1997). The secreted tetramer will bind to a VEGFR3 receptor on an LEC. On the cell surface, it is cooperatively cleaved by CCBE1 and ADAMTS3 (Jeltsch et al. 2014). Our investigation is concerned with the spatiotemporal dynamics of VEGFC on the tissue level, so we are not interested in these events which occur on the cellular level. Therefore, we will not model any cleavage events of VEGFC, intracellular or extracellular, and VEGFC-VEGFR3 binding. It follows that VEGFC denotes the tetramer only in this investigation and it is limited to the interstitial space domain.

We have not discussed the properties of the ECM yet. Its major structural components include different kinds of collagens and glycosaminoglycans (Lutter and Makinen 2014). Collagens make up more than two-thirds of the ECM protein content in many soft tissues (Swartz and Fleury 2007). According to Prockop and Kivirikko (1995), collagen type I is the most abundant protein in humans. More specifically for our study, LECs are mainly surrounded by fibrillar type I collagen in general (Wiig et al. 2010; Paupert et al. 2011). The ECM of an embryo regulates its lymphangiogenic processes in several way (Lutter and Makinen 2014). First, it confers structural support and stability to the embedded cells, tissues, and organs, but it is also a barrier to cell migration. Second, the ECM contains components that can bind to a myriad of cell surface receptors, thus inducing intracellular changes. Third, the ECM can bind to growth factors, thus sequestering them and creating concentration gradients. It is the third function that interests us in this investigation. We will model the transport of VEGFC in the interstitial space domain where it interacts with the ECM. Since LECs are generally surrounded by fibrillar type I collagen, we will treat the ECM as pure collagen I in our model. VEGFC binds to heparan sulphate (Lutter and Makinen 2014), but we do not know whether it binds to collagen I. In order to mimic VEGFC’s interactions with the ECM without modelling heparan sulphate explicitly, we will assume that VEGFC binds to collagen I reversibly in an 1:1 stoichiometric ratio.

An ECM is not inert and undergoes constant remodelling. According to Helm et al. (2007), LECs secrete a protease called matrix metallopeptidase 9 (MMP9) to degrade collagen, thereby rendering their surrounding ECM more conducive to their migration. According to Bruyère et al. (2008), LECs can produce and activate another protease called matrix metallopeptidase 2 (MMP2) to regulate lymphangiogenesis. Commenting on Bruyère et al. (2008), it is argued in Detry et al. (2012) that MMP2 is more important than MMP9. This theory explains lymphangiogenesis in terms of LEC migration through an interstitial collagen I barrier and a collagenolytic pathway driven by MMP2 (Detry et al. 2012). In this investigation, we will consider the production and activation of MMP2 in the LEC domain, as well as the degradation of collagen I by MMP2 in the interstitial space domain. A conceptual model of these MMP2-related events is proposed in Karagiannis and Popel (2004). In this conceptual model, proMMP2, TIMP2, and MT1-MMP act cooperatively to activate proMMP2 to form MMP2. Although MT1-MMP is restricted to the surfaces of LECs, we will disperse the MT1-MMP molecules uniformly in our LEC domain to simplify the mathematics. In our model, the cooperative action occurs in the LEC domain to produce the mature MMP2. However, proMMP2, MMP2, and TIMP2 can all diffuse into the interstitial space domain. In the interstitial space domain, TIMP2 can bind to and inhibit MMP2 reversibly. Karagiannis and Popel (2006) is a mathematical modelling study based on this conceptual model and is an inspiration for our study. It is possible for the interstitial flow to affect the ECM’s composition, either mechanically or by stimulating the LEC to produce or degrade ECM components. Nonetheless, we will assume that the ECM’s behaviour is dominated by collagen I and MMP2 dynamics.

**Fig. 3:**
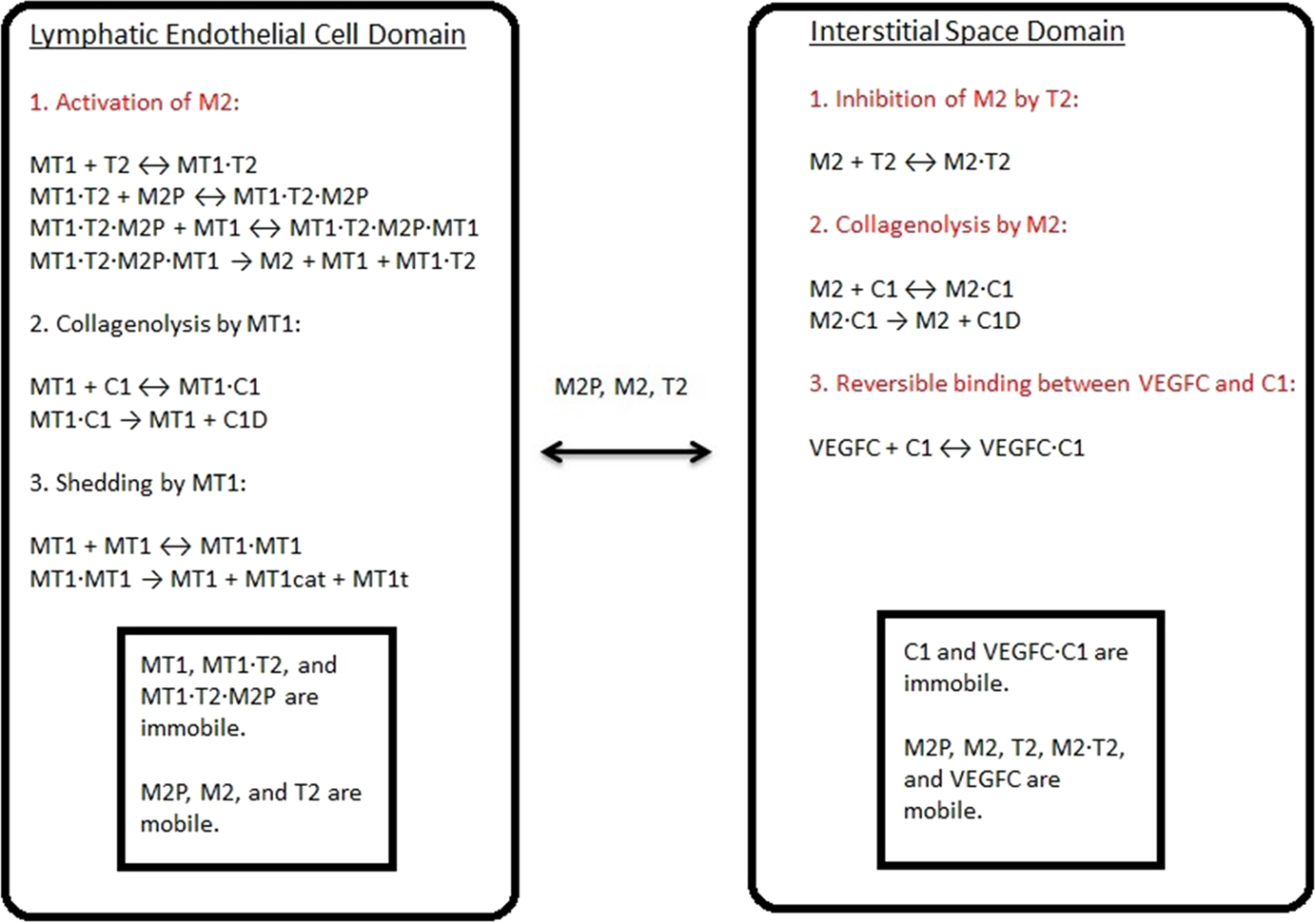
Biochemical reaction network of the model. M2P, proMMP2; M2, MMP2; T2; TIMP2; C1, collagen I; MT1, MT1-MMP. A *dot* between two species means they are complexed together in one molecule. Only proMMP2, MMP2, and TIMP2 are present in both domains and can cross the boundary between them. A mobile species undergoes diffusion and/or convection; an immobile one does not. Only the red events are represented by the mathematical model developed in this paper

Combining the biochemical events described in this subsection, we can construct the overall biochemical reaction network shown in Fig. 3. We will assume that the number of MT1-MMP molecules in the LEC domain is constant, meaning its production rate equals its shedding rate. This assumption allows us to ignore shedding in this study because the shedded species do not interact with the modelled species. MT1-MMP has its own collagenolytic activity too, but it is localised to the LEC domain. In our model, the LEC is a stationary circle devoid of collagen I, so we do not need to model the collagenolytic action of MT1-MMP. Finally, we will not model degraded collagen I explicitly. As far as we are aware, degraded collagen I does not affect lymphangiogenesis.

We will use a set of reaction–diffusion–convection equations to model the spatiotemporal dynamics of the mobile species in the interstitial space. We will use $C_{i}$ (M) to represent the molar concentration of species *i*; *t* (s), time; $D^\mathrm{eff}_{i}$ (µ$\text {m}^{\text {2}}$/s), the effective diffusivity of species *i*; $\omega$, the volume fraction where diffusion occurs; $\varvec{u}$ (µm/s), the velocity from our mathematical model of the interstitial flow; $R^\mathrm{IS}_{i}$ (M/s), the net rate of production of species *i* at a point in the interstitial space. The equation is

7 $$\begin{aligned} \frac{\partial C_{i}}{\partial t}= \varvec{\nabla } \cdot \left( D^\mathrm{eff}_{i}\varvec{\nabla } \left( \frac{C_{i}}{\omega }\right) -\varvec{u}C_{i}\right) + R^\mathrm{IS}_{i}. \end{aligned}$$

Diffusion does not occur in collagen I fibrils or the fluid associated with them. The volume into which a molecular species can diffuse should be based on the specific ‘wet’ weight of collagen I. Denoting the partial specific volume of hydrated collagen I by $v_{{\text {C1h}}}$ ($\text {cm}^{\text {3}}/g$) and the combined mass concentration of free and VEGFC-bound collagen I by $[Cl]_{m}$ (kg/$\text {dm}^{\text {3}}$), we can use a relation from Levick (1987) for $\omega$,

8 $$\begin{aligned} \omega =1-v_{{\text {C1h}}}[Cl]_{m}. \end{aligned}$$

The effective diffusivity of each diffusible species can be calculated by Ogston’s equation (Ogston et al. 1973). Labelling the diffusivity of species *i* in pure interstitial fluid by $D^{\infty }_{i}$ (µ$\text {m}^{\text {2}}$/s), the volume fraction of dry collagen I fibrils (without associated water molecules) by $\nu$, the radius of a collagen I fibril by $r_{f}$ (µm), and the Stokes-Einstein radius of species *i* by $r_{s,i}$ (µm), the equation is

9 $$\begin{aligned} D^\mathrm{eff}_{i}=D^{\infty }_{i}\exp \left( \frac{-\sqrt{\nu }r_{s,i}}{r_{f}}\right) . \end{aligned}$$

Using $v_{\text {C1}}$ ($\text {cm}^{\text {3}}/{\text{ g }}$) to label the partial specific volume of dry collagen I, the equation for the dry volume fraction of collagen I is given in Levick (1987),

10 $$\begin{aligned} \nu =v_{\text {C1}}[Cl]_{m}. \end{aligned}$$

Denoting the Boltzmann constant by $k_\mathrm{B}$ ($1.380648813\times 10^{-23} \hbox {J K}^{-1}$) and temperature by *T* (K), the Stokes–Einstein radius is given in Einstein (1905) as

11 $$\begin{aligned} r_{s,i}=\frac{k_{B}T}{6\pi \mu D^{\infty }_{i}}. \end{aligned}$$

We will use a set of ordinary differential equations to model the temporal dynamics of the immobile species in the interstitial space,

12 $$\begin{aligned} \frac{\partial C_{i}}{\partial t}=R^\mathrm{IS}_{i}. \end{aligned}$$

In the LEC domain, there is neither collagen I nor an interstitial flow. With $R^\mathrm{LEC}_{i}$ (M/s) being the net rate of production of species *i* at a point in the LEC, the governing equations for the mobile species there are reaction–diffusion equations of the form

13 $$\begin{aligned} \frac{\partial C_{i}}{\partial t}=D^{\infty }_{i}\varvec{\nabla }^{2}C_{i}+ R^\mathrm{LEC}_{i}. \end{aligned}$$

In the LEC domain again, the governing equations for the immobile species are a set of ordinary differential equations as follows,

14 $$\begin{aligned} \frac{\partial C_{i}}{\partial t}= R^\mathrm{LEC}_{i}. \end{aligned}$$

The reaction terms, $R^\mathrm{IS}_{i}$ and $R^\mathrm{LEC}_{i}$, can be worked out from the biochemical reaction network in Fig. 3. We will apply mass action kinetics to most reactions. Mass action kinetics assumes that the rate of a reaction is the product of a rate constant and the concentrations of the participating reactants. An exception is the enzymatic degradation of collagen I by MMP2, to which we will employ Michaelis–Menten kinetics. If we assume the complex, MMP2$\cdot$collagen I, is at a steady state, we can approximate collagenolysis by MMP2 as a single second-order kinetic process. This approximation is used and justified in Karagiannis and Popel (2004). We can do the same for the last two steps in the activation of MMP2 by assuming a steady state for the quaternary complex. This approximation is also used and justified in Karagiannis and Popel (2004). There are also some ‘auxiliary’ reactions. proMMP2 and TIMP2 are produced in the LEC. The molecules in the interstitial space are subjected to attacks from enzymes (Gutfreund 1993). Exceptions are collagen I and VEGFC sequestered by collagen I. We are already modelling collagen I degradation by MMP2; sequestration by collagen I protects VEGFC from enzymatic attacks. The reaction terms in the interstitial space, $R^\mathrm{IS}_{i}$, are given in Table 2. The reaction terms in the LEC, $R^\mathrm{LEC}_{i}$, are given in Table 3.

**Table 2:** Reaction terms in the interstitial space

Reaction term	Form	Equations
$R^\mathrm{IS}_{\text {M2P}}$	$-k^\mathrm{deg}_{\text {M2P}}C_{\text {M2P}}$	(7)
$R^\mathrm{IS}_{\text {M2}}$	$-k^\mathrm{on}_{{\text {M2,T2}}}C_{\text {M2}}C_{\text {T2}}+k^\mathrm{off}_{\text {M2,T2}}C_{{\text {M2}}\cdot {\text {T2}}}-k^\mathrm{deg}_{\text {M2}}C_{\text {M2}}$	(7)
$R^\mathrm{IS}_{\text {T2}}$	$-k^\mathrm{on}_{\text {M2,T2}}C_{\text {M2}}C_{\text {T2}}+k^\mathrm{off}_{\text {M2,T2}}C_{{\text {M2}}\cdot {\text {T2}}}-k^\mathrm{deg}_{\text {T2}}C_{\text {T2}}$	(7)
$R^\mathrm{IS}_{{\text {M2}}\cdot {\text {T2}}}$	$k^\mathrm{on}_{\text {M2,T2}}C_{\text {M2}}C_{\text {T2}}-k^\mathrm{off}_{\text {M2,T2}}C_{{\text {M2}}\cdot {\text {T2}}}-k^\mathrm{deg}_{{\text {M2}}\cdot {\text {T2}}}C_{{\text {M2}}\cdot {\text {T2}}}$	(7)
$R^\mathrm{IS}_\mathrm{VC}$	$-k^\mathrm{on}_{\mathrm{VC,C1}}C_\mathrm{VC}C_{\text {C1}}+k^\mathrm{off}_{\mathrm{VC,C1}}C_{\mathrm{VC}\cdot \mathrm{C1}}-k^\mathrm{deg}_\mathrm{VC}C_\mathrm{VC}$	(7)
$R^\mathrm{IS}_{\text {C1}}$	$-\frac{k^\mathrm{cat}_\mathrm{M2,C1}C_{\text {M2}}C_{\text {C1}}}{K^\mathrm{M2,C1}_{M}+C_{\text {C1}}}-k^\mathrm{on}_\mathrm{VC,C1}C_\mathrm{VC}C_{\text {C1}}+k^\mathrm{off}_{\mathrm{VC},C1}C_{\mathrm{VC}\cdot \mathrm{C1}}$	(12)
$R^\mathrm{IS}_{\mathrm{VC}\cdot \mathrm{C1}}$	$k^\mathrm{on}_{\mathrm{VC,C1}}C_\mathrm{VC}C_{\text {C1}}$-$k^\mathrm{off}_{\mathrm{VC,C1}}C_{\mathrm{VC}\cdot \mathrm{C1}}$	(12)

M2 and T2 complex reversibly. VEGFC binds to C1 reversibly. M2 degrades C1 catalytically. M2P, M2, T2, ${\text {M2}}\cdot {\text {T2}}$, and VEGFC degrade in the interstitial space. $k^\mathrm{on}_{\mathrm{VC,C1}}$ ($\hbox {M}^{-1} \hbox {s}^{-1}$) and $k^\mathrm{off}_{\mathrm{VC,C1}}$ ($\hbox {s}^{-1}$) are the binding and unbinding rate constants of VC and C1; $k^\mathrm{on}_{\text {M2,T2}}$ ($\hbox {M}^{-1} \hbox {s}^{-1}$) and $k^\mathrm{off}_{\text {M2,T2}}$ ($\hbox {s}^{-1}$), the binding and unbinding rate constants of M2 and T2; $k^\mathrm{cat}_\mathrm{M2,C1}$ ($\hbox {s}^{-1}$), the turnover number in the degradation of C1 by M2; $K^\mathrm{M2,C1}_{M}$ (M), the Michaelis–Menten constant in the degradation of C1 by M2; $k^\mathrm{deg}_{i}$ ($\hbox {s}^{-1}$), the degradation rate constant of species *i*. M2P, proMMP2; M2, MMP2; T2, TIMP2; VC, VEGFC; C1, collagen I

**Table 3:** Reaction terms in the lymphatic endothelial cell

Reaction term	Form	Equations
$R^\mathrm{LEC}_{\text {M2P}}$	$P_{\text {M2P}}-k^\mathrm{on}_\mathrm{MT1\cdot T2,M2P}C_\mathrm{MT1\cdot T2}C_{\text {M2P}}$	(13)
	+ $k^\mathrm{off}_\mathrm{MT1\cdot T2,M2P}C_\mathrm{MT1\cdot T2\cdot M2P}$	
$R^\mathrm{LEC}_{\text {M2}}$	$k^\mathrm{eff}_\mathrm{act}C_\mathrm{MT1\cdot T2\cdot M2P}C_\mathrm{MT1}$	(13)
$R^\mathrm{LEC}_{\text {T2}}$	$P_{\text {T2}}-k^\mathrm{on}_\mathrm{MT1, T2}C_\mathrm{MT1}C_{\text {T2}}+k^\mathrm{off}_\mathrm{MT1, T2}C_\mathrm{MT1\cdot T2}$	(13)
$R^\mathrm{LEC}_\mathrm{MT1}$	$-\,k^\mathrm{on}_\mathrm{MT1,T2}C_\mathrm{MT1}C_{\text {T2}}+k^\mathrm{off}_\mathrm{MT1,T2}C_\mathrm{MT1\cdot T2}$	(14)
$R^\mathrm{LEC}_\mathrm{MT1 \cdot T2}$	$k^\mathrm{on}_\mathrm{MT1,T2}C_\mathrm{MT1}C_\mathrm{\text {T2}}-k^\mathrm{off}_\mathrm{MT1,T2}C_\mathrm{MT1\cdot T2}$	(14)
	$$-\,k^\mathrm{on}_\mathrm{MT1\cdot T2,M2P}C_\mathrm{MT1\cdot T2}C_\mathrm{\text {M2P}}$$	
	+ $k^\mathrm{off}_\mathrm{MT1\cdot T2,M2P}C_\mathrm{MT1\cdot T2\cdot M2P}$	
	+ $k^\mathrm{eff}_\mathrm{act}C_\mathrm{MT1\cdot T2\cdot M2P}C_\mathrm{MT1}$	
$R^\mathrm{LEC}_\mathrm{MT1 \cdot T2 \cdot M2P}$	$k^\mathrm{on}_\mathrm{MT1\cdot T2,M2P}C_\mathrm{MT1\cdot T2}C_{\text {M2P}}$	(14)
	$$-\,k^\mathrm{off}_\mathrm{MT1\cdot T2,M2P}C_\mathrm{MT1\cdot T2\cdot M2P}$$	
	$$-\,k^\mathrm{eff}_\mathrm{act}C_\mathrm{MT1\cdot T2\cdot M2P}C_\mathrm{MT1}$$	

M2P and T2 are produced at constant rates in the lymphatic endothelial cell. T2 binds to MT1 reversibly. M2P binds to MT1$\cdot$T2 reversibly. MT1 activates the M2P in MT1$\cdot$T2$\cdot$M2P to form M2 and release MT1$\cdot$ T2. $P_{\text {M2P}}$ and $P_{\text {T2}}$ ($\hbox {M s}^{-1}$) are the production rates of M2P and T2 by the lymphatic endothelial cell; $k^\mathrm{on}_\mathrm{MT1,T2}$ ($\hbox {M}^{-1} \hbox {s}^{-1}$) and $k^\mathrm{off}_\mathrm{MT1,T2}$ ($\hbox {s}^{-1}$), the binding and unbinding rate constants of MT1 and T2; $k^\mathrm{on}_\mathrm{MT1\cdot T2,M2P}$ ($\hbox {M}^{-1} \hbox {s}^{-1}$) and $k^\mathrm{off}_\mathrm{MT1\cdot T2,M2P}$ ($\hbox {s}^{-1}$), the binding and unbinding rate constants of $\text {MT1}\cdot {\text {T2}}$ and M2P; $k^\mathrm{eff}_\mathrm{act}$ ($\hbox {M}^{-1} \hbox {s}^{-1}$), the activation rate constant of M2. M2P, proMMP2; M2, MMP2; T2, TIMP2; MT1, MT1-MMP

On the four outer boundaries of the geometry and the vessel surfaces, we will impose no-flux boundary conditions for each mobile species. Our choices are justified as follows. First, there is no evidence that zebrafish lose the modelled molecules through their skin. While we could model this hypothetical mechanism, our model will not be less descriptive without it. Second, to the best of our knowledge, there is no evidence that the modelled molecules can enter the blood vessels. If they do pass through the vessel surfaces, we have no idea how much is filtered by the lining cells which may have binding receptors, for example. Anyway, they will simply degrade inside the vessels, so we will not model this hypothetical and poorly defined phenomenon. An exception is the flux of VEGFC from the DA surface into the interstitial space. VEGFC is expressed in the hypochord, the dorsal aorta, and the ventral mesenchyme of a zebrafish at 48 HPF (Hogan et al. 2009). The high pressure in the DA means convection is most significant around it. We will place the source of VEGFC on the DA’s surface. This arrangement will retain the essences of VEGFC transport in zebrafish embryos and keep the model simple simultaneously. We should remind ourselves that VEGFC is not released from the blood inside the DA. Its production by a part of the DA’s wall is independent of that elsewhere on the DA, so its release rate does not change along the DA.

On the four outer boundaries, the no-flux boundary conditions for proMMP2, MMP2, TIMP2, MMP2$\cdot$TIMP2, and VEGFC are given by

15 $$\begin{aligned} \varvec{n} \cdot \left[ D^\mathrm{eff}_{i}\varvec{\nabla }\left( \frac{C_{i}}{\omega }\right) -\varvec{u}C_{i}\right] =0 \qquad \varvec{x} \in \partial \varOmega _{x,y}. \end{aligned}$$

On the PCV and the DLAV, they are

16 $$\begin{aligned} \varvec{n} \cdot \left[ D^\mathrm{eff}_{i}\varvec{\nabla }\left( \frac{C_{i}}{\omega }\right) -\varvec{u}C_{i}\right]= & {} 0 \qquad \varvec{x} \in \partial \varOmega _\mathrm{PCV} \; \text {and} \end{aligned}$$

17 $$\begin{aligned} \varvec{n} \cdot \left[ D^\mathrm{eff}_{i}\varvec{\nabla }\left( \frac{C_{i}}{\omega }\right) -\varvec{u}C_{i}\right]= & {} 0 \qquad \varvec{x} \in \partial \varOmega _\mathrm{DLAV}. \end{aligned}$$

On the DA, the boundary conditions for the mobile species except VEGFC are

18 $$\begin{aligned} \varvec{n} \cdot \left[ D^\mathrm{eff}_{i}\varvec{\nabla }\left( \frac{C_{i}}{\omega }\right) -\varvec{u}C_{i}\right] =0 \qquad \varvec{x} \in \partial \varOmega _\mathrm{DA}. \end{aligned}$$

For VEGFC, with $R^\mathrm{DA}_\mathrm{VC}$ (mol µ$\hbox {m}^{-2} \hbox {s}^{-1}$) being the release rate of VEGFC on the surface of the DA, the constant flux is

19 $$\begin{aligned} -\varvec{n} \cdot \left[ -D^\mathrm{eff}_\mathrm{VC}\varvec{\nabla }\left( \frac{C_\mathrm{VC}}{\omega }\right) +\varvec{u}C_\mathrm{VC}\right] =R^\mathrm{DA}_\mathrm{VC} \qquad \varvec{x} \in \partial \varOmega _\mathrm{DA}. \end{aligned}$$

On the LEC’s surface, we will impose continuity conditions on the species which can cross the boundary between the LEC and interstitial space domains. We will denote the LEC surface seen from the interstitial space by $\partial \varOmega _\mathrm{LEC/IS+}$. Seen from the LEC domain, the surface is $\partial \varOmega _\mathrm{LEC/IS-}$. Continuity applies to proMMP2, MMP2, and TIMP2 as follows,

20 $$\begin{aligned} \varvec{n} \cdot \left[ D^\mathrm{eff}_{i}\varvec{\nabla }\left( \frac{C_{i}}{\omega }\right) -\varvec{u}C_{i}\right] \arrowvert _{\partial \varOmega _\mathrm{LEC/IS+}}= & {} -\varvec{n} \cdot \left( D^{\infty }_{i}\varvec{\nabla }C_{i}\right) \arrowvert _{\partial \varOmega _\mathrm{LEC/IS-}} \; \text {and} \end{aligned}$$

21 $$\begin{aligned} C_{i}\arrowvert _{\partial \varOmega _\mathrm{LEC/IS+}}= & {} C_{i}\arrowvert _{\partial \varOmega _\mathrm{LEC/IS-}}. \end{aligned}$$

On the LEC surface seen from the interstitial space, we will apply no-flux conditions on MMP2$\cdot$TIMP2 and VEGFC,

22 $$\begin{aligned} \varvec{n} \cdot \left[ D^\mathrm{eff}_{i}\varvec{\nabla }\left( \frac{C_{i}}{\omega }\right) -\varvec{u}C_{i}\right] = 0 \qquad \varvec{x} \in \partial \varOmega _\mathrm{LEC/IS+}. \end{aligned}$$

We also need a set of initial concentrations. In the LEC domain, we will assume that only MT1-MMP is present at $t=0$; in the interstitial space, we will assume that only collagen I is present at $t=0$. We will label the initial concentrations of these species by $C_{{\text {MT1}},0}$ and $C_{{\text {C1}},0}$, respectively.

### Connection of Extracellular Matrix Remodelling to Interstitial Flow

The interstitial flow is dependent on the composition of the interstitial space. Specifically, it depends on the dynamics of the ECM. This is obvious from Eq. (1), where $\kappa$ is the specific hydraulic conductivity of the ECM. As the ECM remodels, this conductivity will also change, thereby affecting the interstitial flow.

We will define $\kappa '$ ($\hbox {cm}^4\, \hbox {s}^{-1} \hbox {dyn}^{-1}$) as the hydraulic conductivity of the ECM and $[{\text {Collagen}}\;{\text {I}}]$ as the mass fraction of free and VEGFC-bound collagen I in the interstitial space. Using the experimental data presented in Levick (1987), we can relate the two by

23 $$\begin{aligned} \log \kappa '=-2.70\log \left[ \mathrm{Collagen\;I}\right] -14.18. \end{aligned}$$

With $M_{\text {C1}}$ ($\hbox {kg mol}^{-1}$) being the molar mass of collagen I, $[\mathrm{Collagen\;I}]$ is given by

24 $$\begin{aligned}{}[{\text {Collagen}}\;{\text {I}}]=\frac{M_{\text {C1}}(C_{\text {C1}}+C_{\mathrm{VC}\cdot \mathrm{C1}})}{1\;\text {kg}\;\text {dm}^{-3}}. \end{aligned}$$

Equation (24) assumes a density of 1 kg $\hbox {dm}^{-3}$ for the interstitial space. It is also assumed that the combined mass of the interstitial fluid and the ECM in the interstitial space is conserved. When collagen I degrades, the products remain in the interstitial space, so the mass of a region is fixed at 1 kg $\hbox {dm}^{-3}$ multiplied by the volume of the region. The experiments cited in Levick (1987) were carried out using a reference fluid with a dynamic viscosity of 1 cP. We can therefore convert the hydraulic conductivity to the specific hydraulic conductivity by $\kappa =\kappa '\times 10^{-2} \text {dyn s}\;\text {cm}^{{-2}}$.

### Parametrisation

We will begin our parametrisation with the interstitial flow component of the model. We need the pressures inside the three blood vessels, their permeabilities, as well as the properties of the interstitial fluid and the ECM. Table 4 summarises these parameters whose origins are explained below.

**Table 4:** Parameters of the interstitial flow component of the mathematical model

Parameter	Definition	Value	Reference
$P^\mathrm{DA}$	DA pressure	0.1844 mmHg	Hu et al. (2000)
$P^\mathrm{DLAV}$	DLAV pressure	0 mmHg	Assumed
$P^\mathrm{PCV}$	PCV pressure	0 mmHg	Assumed
$L_\mathrm{DA}$	DA permeability	$7.20\times 10^{6}\,\hbox {cm}\,\hbox {s}^{-1}\,\hbox {cm}\,\text {H}_{\text {2}}\text {O}^{-1}$ cm $\hbox {s}^{-1}$ cm$\text {H}_{\text {2}}\text {O}^{-1}$	Jain (1987)
$L_\mathrm{DLAV}$	DLAV permeability	$7.20\times 10^{6}\,\hbox {cm}\,\hbox {s}^{-1} \hbox {cm}\text {H}_{\text {2}}\text {O}^{-1}$	Jain (1987)
$L_\mathrm{PCV}$	PCV permeability	$7.20\times 10^{6}$ cm $\hbox {s}^{-1}$ cm$\text {H}_{\text {2}}\text {O}^{-1}$	Jain (1987)
$M_{\text {C1}}$	Molar mass of collagen I	300 kg $\hbox {mol}^{-1}$	Karagiannis and Popel (2006)
$\mu$	IF dynamic viscosity	1.200 cP	Swartz and Fleury (2007)
$\rho$	IF density	1025 kg $\hbox {m}^{-3}$	Frcitas (1998)

*DA* dorsal aorta, *PCV* posterior cardinal vein, *DLAV* dorsal longitudinal anastomotic vessel, *IF* interstitial fluid

In Hu et al. (2000), the DA peak systolic and end-diastolic pressures of zebrafish embryos are related to their wet body weights. At 48 HPF, the wet body weight is 0.714 mg. Ignoring the pulsating nature of blood flow, we will average the measured peak systolic (0.2433 mmHg) and end-diastolic (0.1255 mmHg) pressures corresponding to this wet body weight. This gives an estimated DA pressure of 0.1844 mmHg relative to the pressure outside the embryo. The PCV and the DLAV are parts of a closed and pumped blood circulatory system, so they must have higher pressures than the embryo’s unpumped surrounding. However, in the absence of data about pressure drops in the circulatory system, back-of-the-envelope estimates are unlikely to be accurate. For the sake of simplicity, we will just set the PCV and DLAV pressures to zero. The DA pressure must drop along the vessel from the zebrafish’s heart. Our geometry includes a slice of the DA only, so the pressure drop does not show up in the model. It is not important either because the lymphangiogenic processes in Fig. 1 do not occur along the anterior–posterior axis.

We have already decided to use one vascular permeability for the three blood vessels. We will rely on Jain (1987) for this parameter. The reported measurements are concerned with species ranging from Guinea pigs to frogs. Although zebrafish is not among these species, we can use the permeability for frog skeletal muscles, $7.2\times 10^{6}$ cm $\hbox {s}^{-1}$ cm$\text {H}_{\text {2}}\text {O}^{-1}$. The justification is that both frogs and zebrafish are cold-blooded.

The specific hydraulic conductivity of the ECM is given by the relation in Sect. 2.4. However, we need $M_{\text {C1}}$ in Eq. (24). The model in Karagiannis and Popel (2006) uses a molecular weight of around 300 kDa for a collagen I fibril, which is equivalent to a molar mass of 300 kg $\hbox {mol}^{-1}$.

The interstitial fluid contains roughly 40% of the protein concentration of blood plasma (Swartz and Fleury 2007). Their similarities in composition allow us to use the parameter values for blood plasma. At $37\,^{\circ }\mathrm{C}$, the dynamic viscosity of blood plasma is 1.2 cP (Swartz and Fleury 2007) and its density is 1025 $\hbox {kg/m}^3$ (Frcitas 1998).

We will turn our attention to the reaction–diffusion–convection equation and its reduced forms next. Their parameters can be categorised into transport and kinetic parameters.

In order to calculate the volume fraction where diffusion occurs using Eq. (8) and the effective diffusivity of a species using Eq. (9), we need several parameters. We will assume a temperature of 298 K. A collagen I fibril is approximately 300 nm long and 4 nm in diameter, so $r_{f}$ is 2 nm (Karagiannis and Popel 2006). We can also find the required $D^{\infty }_{i}$ values in Karagiannis and Popel (2006) and Berk et al. (1993). The partial specific volume of dry collagen I and that of hydrated collagen I, $v_{\text {C1}}$ and $v_{{\text {C1h}}}$, are 0.75 and 1.89 $\hbox {cm}^3/\hbox {g}$ (Levick 1987). These parameters are summarised in Table 5.

**Table 5:** Transport parameters in the reaction–diffusion–convection equation and its simplified forms

Parameter	Definition	Value	References
$D^{\infty }_\mathrm{VC}$	Diffusivity of VC	$5.01\times 10^{-7}$ $\hbox {cm}^2/\hbox {s}$	Berk et al. (1993)
$D^{\infty }_{\text {M2}}$	Diffusivity of M2	$0.85\times 10^{-6}$ $\hbox {cm}^2/\hbox {s}$	Karagiannis and Popel (2006)
$D^{\infty }_{\text {M2P}}$	Diffusivity of M2P	$0.80\times 10^{-6}$ $\hbox {cm}^2/\hbox {s}$	Karagiannis and Popel (2006)
$D^{\infty }_{{\text {M2}}\cdot {\text {T2}}}$	Diffusivity of M2$\cdot$T2	$0.75\times 10^{-6}$ $\hbox {cm}^2/\hbox {s}$	Karagiannis and Popel (2006)
$D^{\infty }_{\text {T2}}$	Diffusivity of T2	$1.10\times 10^{-6}$ $\hbox {cm}^2/\hbox {s}$	Karagiannis and Popel (2006)
$r_{f}$	Radius of a C1 fibril	2 nm	Karagiannis and Popel (2006)
$v_{\text {C1}}$	Specific volume of dry C1	0.75 $\hbox {cm}^3/\hbox {s}$	Levick (1987)
$v_{{\text {C1h}}}$	Specific volume of hydrated C1	1.89 $\hbox {cm}^3/\hbox {s}$	Levick (1987)
T	Temperature	298 K	Assumed

M2P, proMMP2; M2, MMP2; T2, TIMP2; VC, VEGFC; C1, collagen I

The majority of our kinetic parameters about ECM remodelling are from Karagiannis and Popel (2006, 2004). Their sources are various experimental studies. We are unaware of any data on VEGFC–collagen I interactions. In fact, we do not even know whether VEGFC binds to collagen I. We must rely on other data. In Köhn-Luque et al. (2013), there are reports of experimentally estimated parameters on the interactions between VEGF (related to but different from VEGFC) and various ECM molecules like fibronectin and heparan sulphate proteoglycans. We will use the general degradation rate constant used in Hashambhoy et al. (2011). For the production rates of proMMP2 and TIMP2, we will use the secretion rates of general MMP and TIMP by endothelial cells, used in Vempati et al. (2010). These estimates are in molecules/cell/h, but we can convert them to M $\hbox {s}^{-1}$ using the known LEC diameter of 10 $\upmu \hbox {m}$. We are unaware of any data on the production rate of VEGFC. Therefore, we will use the secretion rate of VEGF by endothelial cells, estimated in Hashambhoy et al. (2011). These parameters are summarised in Table 6.

**Table 6:** Kinetic parameters in the reaction–diffusion–convection equation and its simplified forms

Parameter	Value	References
$k^\mathrm{on}_{\mathrm{VC,C1}}$	$3.60\times 10^{4}$ $\hbox {M}^{-1} \hbox {s}^{-1}$	Köhn-Luque et al. (2013)
$k^\mathrm{off}_{\mathrm{VC,C1}}$	$3.60\times 10^{-3}$ $\hbox {s}^{-1}$	Köhn-Luque et al. (2013)
$k^\mathrm{on}_{\text {M2,T2}}$	$5.90\times 10^{6}$ $\hbox {M}^{-1}$ $\hbox {s}^{-1}$	Karagiannis and Popel (2004)
$k^\mathrm{off}_{\text {M2,T2}}$	6.30 $\hbox {s}^{-1}$	Karagiannis and Popel (2004)
$k^\mathrm{on}_\mathrm{MT1,T2}$	$3.54\times 10^{6}$ $\hbox {M}^{-1}$ $\hbox {s}^{-1}$	Toth et al. (2002)
$k^\mathrm{off}_\mathrm{MT1,T2}$	$2\times 10^{-4}$ $\hbox {s}^{-1}$	Toth et al. (2002)
$k^\mathrm{on}_\mathrm{MT1\cdot T2,M2P}$	$0.14\times 10^{6}$ $\hbox {M}^{-1}$ $\hbox {s}^{-1}$	Karagiannis and Popel (2004)
$k^\mathrm{off}_\mathrm{MT1\cdot T2,M2P}$	$4.70\times 10^{-3}$ $\hbox {s}^{-1}$	Karagiannis and Popel (2004)
$k^\mathrm{eff}_\mathrm{act}$	$2.80\times 10^{3}$ $\hbox {M}^{-1}$ $\hbox {s}^{-1}$	Karagiannis and Popel (2004)
$k^\mathrm{cat}_\mathrm{M2,C1}$	$4.50\times 10^{-3}$ $\hbox {s}^{-1}$	Karagiannis and Popel (2004)
$K^\mathrm{M2,C1}_{M}$	$8.50\times 10^{-6}$ M	Karagiannis and Popel (2004)
$k^\mathrm{deg}_\mathrm{VC}$	$10^{-4}$ $\hbox {s}^{-1}$	Hashambhoy et al. (2011)
$k^\mathrm{deg}_{\text {M2}}$	$10^{-4}$ $\hbox {s}^{-1}$	Hashambhoy et al. (2011)
$k^\mathrm{deg}_{\text {M2P}}$	$10^{-4}$ $\hbox {s}^{-1}$	Hashambhoy et al. (2011)
$k^\mathrm{deg}_{{\text {M2}}\cdot {\text {T2}}}$	$10^{-4}$ $\hbox {s}^{-1}$	Hashambhoy et al. (2011)
$k^\mathrm{deg}_{\text {T2}}$	$10^{-4}$ $\hbox {s}^{-1}$	Hashambhoy et al. (2011)
$P_{\text {M2P}}$	$2.64\times 10^{-8}$ M $\hbox {s}^{-1}$	Vempati et al. (2010)
$P_{\text {T2}}$	$1.54\times 10^{-10}$ M $\hbox {s}^{-1}$	Vempati et al. (2010)
$R^\mathrm{DA}_\mathrm{VC}$	$1.65\times 10^{-17}$ mol $\hbox {dm}^{-2}$ $\hbox {s}^{-1}$	Hashambhoy et al. (2011)

$k^\mathrm{on}_{i,j}$ means the binding rate constant of species *i* and *j*; $k^\mathrm{off}_{i,j}$, their unbinding rate constant; $k^\mathrm{eff}_\mathrm{act}$, the activation rate constant of M2; $k^\mathrm{cat}_\mathrm{M2,C1}$, the turnover number in the degradation of C1 by M2; $K^\mathrm{M2,C1}_{M}$, the Michaelis–Menten constant in the degradation of C1 by M2; $k^\mathrm{deg}_{i}$, the degradation rate constant of species *i*; $P_{i}$, the production rate of species *i*; $R^\mathrm{DA}_\mathrm{VC}$, the production rate of VC on the surface of the dorsal aorta

M2P, proMMP2; M2, MMP2; T2, TIMP2; VC, VEGFC; C1, collagen I; MT1, MT1-MMP

Finally, we need the initial concentration of MT1-MMP in the LEC and that of collagen I in the interstitial space. $C_{{\text {MT1}},0}$ is unavailable for endothelial cells. However, a value based on other cell types, 180,000 molecules/cell or $5.71\times 10^{-7}$ M, is in Karagiannis and Popel (2006). In adult tissues, the concentration of collagen I ranges from $1.76\times 10^{-4}$ to $5.29\times 10^{-4}$ M (Levick 1987; Karagiannis and Popel 2006). We will use the midpoint of this range, so $C_{{\text {C1}},0}$ is $3.50\times 10^{-4}$ M for adult tissues. The collagen content of frog embryos and larvae ranges from $4.51\times 10^{-7}$ M to $2.73\times 10^{-6}$ M (Edds Jr 1958). We will use the midpoint of this range, $1.59\times 10^{-6}$ M.

### Nondimensionalisation

Nondimensionalisation reduces the number of parameters in a mathematical model, gives us insights into the model in terms of its key parameters and characteristic properties, and identifies any mathematical techniques for approximation like limiting cases. We need the characteristic scales of the model in order to nondimensionalise it. To obtain the nondimensionalised spatial coordinates, $\tilde{\varvec{x}}=\frac{\varvec{x}}{L}$, we need the length scale, *L*; we will use the largest dimension of the geometry, 434 µm. We will use $P^\mathrm{DA}=0.1844$ mmHg to scale the pressure field, $\tilde{P}=\frac{P}{P^\mathrm{DA}}$. Because we are interested in the period from 36 to 48 HPF, we will use a time scale, $\tau$, of 12 h. The nondimensionalised time is therefore $\tilde{t}=\frac{t}{\tau }$. Velocity is scaled like $\tilde{\varvec{u}}=\frac{\varvec{u}}{U}$. The concentrations are nondimensionalised like $\tilde{C_{i}}=\frac{C_{i}}{C_{i,s}}$. Since we are not modelling collagen I synthesis, its initial concentration is also its highest possible concentration. Therefore, we will use the initial concentration of collagen I as its scale, $C_\mathrm{C1,s}=C_{{\text {C1}},0}$. We will use the adult value, $C_{{\text {C1}},0}=3.50\times 10^{-4}$ M. The concentrations of MT1-MMP and its two complexes will always add up to the initial concentration of MT1-MMP, $C_{{\text {MT1}},0}=5.71\times 10^{-7}$ M. We will use this value for $C_\mathrm{MT1,s}$, $C_\mathrm{MT1 \cdot T2,s}$, and $C_\mathrm{MT1 \cdot T2 \cdot M2P,s}$. We will determine the velocity scale, *U* (µm/s), and the remaining concentration scales while nondimensionalising the model. The scales are summarised in Table 7. Nondimensionalising the model by these scales, we will obtain a model parametrised by the dimensionless groups in Tables 8 and 9. Below are the details of how nondimensionalisation is carried out for our mathematical model.

**Table 7:** Scales used for nondimensionalisation

Scale	Description	Value
$C_\mathrm{C1,s}$	Concentration scale for C1	$3.50\times 10^{-4}$ M
$C_\mathrm{VC,s}$	Concentration scale for VC	$1.64\times 10^{-10}$ M
$C_\mathrm{VC \cdot C1,s}$	Concentration scale for VC$\cdot$C1	$8.93\times 10^{-5}$ M
$C_\mathrm{M2,s}$	Concentration scale for M2	$3.94\times 10^{-5}$ M
$C_\mathrm{M2P,s}$	Concentration scale for M2P	$1.14\times 10^{-3}$ M
$C_\mathrm{M2 \cdot T2,s}$	Concentration scale for M2$\cdot$T2	$6.68\times 10^{1}$ M
$C_\mathrm{MT1,s}$	Concentration scale for MT1	$5.71\times 10^{-7}$ M
$C_\mathrm{MT1 \cdot T2,s}$	Concentration scale for MT1$\cdot$T2	$5.71\times 10^{-7}$ M
$C_\mathrm{MT1 \cdot T2 \cdot M2P,s}$	Concentration scale for MT1$\cdot$T2$\cdot$M2P	$5.71\times 10^{-7}$ M
$C_\mathrm{T2,s}$	Concentration scale for T2	$6.65\times 10^{-6}$ M
*L*	Length scale	434 $\upmu \hbox {m}$
$P^\mathrm{DA}$	Pressure scale and DA pressure	0.1844 mmHg
$\tau$	Time scale	43200 s
*U*	Velocity scale	$1.371\times 10^{-4}$ $\upmu \hbox {m}$/s

M2P, proMMP2; M2, MMP2; T2, TIMP2; VC, VEGFC; C1, collagen 1; MT1, MT1-MMP

**Table 8:** Dimensionless parameters in the nondimensionalised interstitial flow and reaction–diffusion–convection equation

Parameter	Form	Value
$\alpha$	Constant	−2.70
$\eta _{1}$	$\frac{\mu U L}{P^\mathrm{DA}\beta (M_{\text {C1}}C_\mathrm{C1,s})^{\alpha }}$	1
$\eta _{2}$	$\frac{C_{{\text {VC}} \cdot {\text {C1,s}}}}{C_\mathrm{C1,s}}$	0.255
$\eta _{3}$	$\frac{\mu U}{L P^\mathrm{DA}}$	$1.542\times 10^{-11}$
$\eta _\mathrm{DA}$	$\frac{L_\mathrm{DA}P^{DA}}{U}$	$1.317\times 10^{14}$
$\eta _\mathrm{PCV}$	$\frac{L_\mathrm{PCV}P^\mathrm{DA}}{U}$	$1.317\times 10^{14}$
$\eta _\mathrm{DLAV}$	$\frac{2L_\mathrm{DLAV}P^\mathrm{DA}}{U}$	$2.634\times 10^{14}$
$\lambda _\mathrm{1,VC}$	$\frac{D^{\infty }_\mathrm{VC}\tau }{L^{2}}$	$1.15\times 10^{1}$
$\lambda _\mathrm{1,M2}$	$\frac{D^{\infty }_{\text {M2}}\tau }{L^{2}}$	$1.95\times 10^{1}$
$\lambda _\mathrm{1,M2P}$	$\frac{D^{\infty }_{\text {M2P}}\tau }{L^{2}}$	$1.83\times 10^{1}$
$\lambda _\mathrm{1,M2 \cdot T2}$	$\frac{D^{\infty }_{\text {M2}}\cdot {\text {T2}}\tau }{L^{2}}$	$1.72\times 10^{1}$
$\lambda _\mathrm{1,T2}$	$\frac{D^{\infty }_{\text {T2}}\tau }{L^{2}}$	$2.52\times 10^{1}$
$\lambda _\mathrm{2,VC}$	$\frac{k_\mathrm{B}T}{6\pi \mu D^{\infty }_\mathrm{VC} r_{f}}$	1.81
$\lambda _\mathrm{2,M2}$	$\frac{k_\mathrm{B}T}{6\pi \mu D^{\infty }_{\text {M2}} r_{f}}$	1.07
$\lambda _\mathrm{2,M2P}$	$\frac{k_\mathrm{B}T}{6\pi \mu D^{\infty }_{\text {M2P}} r_{f}}$	1.14
$\lambda _\mathrm{2,M2 \cdot T2}$	$\frac{k_\mathrm{B}T}{6\pi \mu D^{\infty }_{\text {M2}}\cdot {\text {T2}} r_{f}}$	1.21
$\lambda _\mathrm{2,T2}$	$\frac{k_\mathrm{B}T}{6\pi \mu D^{\infty }_{\text {T2}} r_{f}}$	$8.26\times 10^{-1}$
$\lambda _\mathrm{3}$	$v_{\text {C1}}M_{\text {C1}}C_\mathrm{C1,s}$	$7.88\times 10^{-2}$
$\lambda _{4}$	$v_{\text {C1}}M_{\text {C1}}C_\mathrm{VC \cdot C1,s}$	$2.01\times 10^{-2}$
$\lambda _{5}$	$v_{{\text {C1h}}}M_{\text {C1}}C_\mathrm{C1,s}$	$1.98\times 10^{-1}$
$\lambda _{6}$	$v_{{\text {C1h}}}M_{\text {C1}}C_\mathrm{VC \cdot C1,s}$	$5.06\times 10^{-2}$
$\lambda _{7}$	$\frac{U\tau }{L}$	$1.36\times 10^{-2}$

*DA* dorsal aorta, *PCV* posterior cardinal vein, *DLAV* dorsal longitudinal anastomotic vessel

M2P, proMMP2; M2, MMP2; T2, TIMP2; VC, VEGFC; C1, collagen I

**Table 9:** Dimensionless parameters in the nondimensionalised reaction terms

Parameter	Form	Value
$\lambda _{8}$	$\frac{P_{\text {M2P}}\tau }{C_\mathrm{M2P,s}}$	1
$\lambda _{9}$	$k^\mathrm{on}_\mathrm{MT1 \cdot T2,M2P}\tau C_\mathrm{MT1 \cdot T2,s}$	$3.45\times 10^{3}$
$\lambda _{10}$	$\frac{k^\mathrm{off}_\mathrm{MT1 \cdot T2,M2P}\tau C_\mathrm{MT1 \cdot T2 \cdot M2P,s}}{C_\mathrm{M2P,s}}$	$1.02\times 10^{-1}$
$\lambda _{11}$	$k^\mathrm{deg}_{\text {M2P}} \tau$	4.32
$\lambda _{12}$	$\frac{k^\mathrm{eff}_\mathrm{act}\tau C_\mathrm{MT1\cdot T2\cdot M2P,s}C_\mathrm{MT1,s}}{C_\mathrm{M2,s}}$	1
$\lambda _{13}$	$k^\mathrm{on}_{\text {M2,T2}}\tau C_\mathrm{T2,s}$	$1.69\times 10^{6}$
$\lambda _{14}$	$\frac{k^\mathrm{off}_{\text {M2,T2}}\tau C_{{\text {M2}}\cdot {\text {T2,s}}}}{C_\mathrm{M2,s}}$	$4.61\times 10^{11}$
$\lambda _{15}$	$k^\mathrm{deg}_{\text {M2}}\tau$	4.32
$\lambda _{16}$	$\frac{P_{\text {T2}}\tau }{C_\mathrm{T2,s}}$	1
$\lambda _{17}$	$k^\mathrm{on}_\mathrm{MT1, T2}\tau C_\mathrm{MT1,s}$	$8.73\times 10^{4}$
$\lambda _{18}$	$\frac{k^\mathrm{off}_\mathrm{MT1, T2}\tau C_\mathrm{MT1\cdot T2,s}}{C_\mathrm{T2,s}}$	$7.42\times 10^{-1}$
$\lambda _{19}$	$k^\mathrm{on}_{\text {M2,T2}}\tau C_\mathrm{M2,s}$	$1\times 10^{7}$
$\lambda _{20}$	$\frac{k^\mathrm{off}_{\text {M2,T2}}\tau C_{{\text {M2}}\cdot {\text {T2,s}}}}{C_\mathrm{T2,s}}$	$2.73\times 10^{12}$
$\lambda _{21}$	$k^\mathrm{deg}_{\text {T2}}\tau$	4.32
$\lambda _{22}$	$\frac{k^\mathrm{on}_{\text {M2,T2}}\tau C_\mathrm{M2,s}C_\mathrm{T2,s}}{C_{{\text {M2}}\cdot {\text {T2,s}}}}$	1
$\lambda _{23}$	$k^\mathrm{off}_{\text {M2,T2}}\tau$	$2.72\times 10^{5}$
$\lambda _{24}$	$k^\mathrm{deg}_{{\text {M2}}\cdot {\text {T2}}}\tau$	4.32
$\lambda _{25}$	$k^\mathrm{on}_\mathrm{VC,C1}\tau C_\mathrm{C1,s}$	$5.44\times 10^{5}$
$\lambda _{26}$	$\frac{k^\mathrm{off}_\mathrm{VC,C1}\tau C_\mathrm{VC\cdot C1,s}}{C_\mathrm{VC,s}}$	$8.47\times 10^{7}$
$\lambda _{27}$	$k^\mathrm{deg}_\mathrm{VC}\tau$	4.32
$\lambda ^\mathrm{DA}_\mathrm{VC}$	$\frac{R^\mathrm{DA}_\mathrm{VC} \tau }{C_\mathrm{VC,s}L}$	1
$\lambda _{28}$	$k^\mathrm{cat}_\mathrm{M2,C1}\tau C_\mathrm{M2,s}$	$7.66\times 10^{-3}$
$\lambda _{29}$	$k^\mathrm{on}_{\mathrm{VC,C1}}\tau C_{\mathrm{VC,s}}$	$2.55\times 10^{-1}$
$\lambda _{30}$	$\frac{k^\mathrm{off}_{\mathrm{VC,C1}}\tau C_\mathrm{VC\cdot C1,s}}{C_\mathrm{C1,s}}$	$3.97\times 10^{1}$
$\lambda _{31}$	$\frac{k^\mathrm{on}_{\mathrm{VC,C1}}\tau C_{\mathrm{VC,s}}C_\mathrm{C1,s}}{C_\mathrm{VC\cdot C1,s}}$	1
$\lambda _{32}$	$k^\mathrm{off}_{\mathrm{VC,C1}}\tau$	$1.56\times 10^{2}$
$\lambda _{33}$	$k^\mathrm{on}_\mathrm{MT1,T2}\tau C_\mathrm{T2,s}$	$1.02\times 10^{6}$
$\lambda _{34}$	$\frac{k^\mathrm{off}_\mathrm{MT1,T2} \tau C_\mathrm{MT1\cdot T2,s}}{C_\mathrm{MT1,s}}$	8.64
$\lambda _{35}$	$\frac{k^\mathrm{on}_\mathrm{MT1,T2} \tau C_\mathrm{MT1,s} C_\mathrm{T2,s}}{C_\mathrm{MT1\cdot T2,s}}$	$1.02\times 10^{6}$
$\lambda _{36}$	$k^\mathrm{off}_\mathrm{MT1,T2} \tau$	8.64
$\lambda _{37}$	$k^\mathrm{on}_\mathrm{MT1 \cdot T2,M2P} \tau C_\mathrm{M2P,s}$	$6.89\times 10^{6}$
$\lambda _{38}$	$\frac{k^\mathrm{off}_\mathrm{MT1 \cdot T2,M2P} \tau C_\mathrm{MT1 \cdot T2 \cdot M2P,s}}{C_\mathrm{MT1\cdot T2,s}}$	$2.03\times 10^{2}$
$\lambda _{39}$	$\frac{k^\mathrm{eff}_\mathrm{act} \tau C_\mathrm{MT1 \cdot T2 \cdot M2P,s}C_\mathrm{MT1,s}}{C_\mathrm{MT1\cdot T2,s}}$	$6.91\times 10^{1}$
$\lambda _{40}$	$\frac{k^\mathrm{on}_\mathrm{MT1 \cdot T2,M2P} \tau C_\mathrm{MT1 \cdot T2,s}C_\mathrm{M2P,s}}{C_\mathrm{MT1 \cdot T2 \cdot M2P,s}}$	$6.89\times 10^{6}$
$\lambda _{41}$	$k^\mathrm{off}_\mathrm{MT1 \cdot T2,M2P}\tau$	$2.03\times 10^{2}$
$\lambda _{42}$	$k^\mathrm{eff}_\mathrm{act} \tau C_\mathrm{MT1,s}$	$6.91\times 10^{1}$

It should be noted that $\lambda _{28}$ is not really dimensionless and is in M, but the term $\frac{\lambda _{28}\tilde{C}_{\text {M2}}\tilde{C}_{\text {C1}}}{K^\mathrm{M2,C1}_{M}+C_\mathrm{C1,s}\tilde{C}_{\text {C1}}}$ is dimensionless because the denominator is also in M

M2P, proMMP2; M2, MMP2; T2, TIMP2; VC, VEGFC; C1, collagen 1; MT1, MT1-MMP; *DA* dorsal aorta

First, we will nondimensionalise the interstitial flow equations. In this study, a tilde represents a nondimensionalised variable, for example, $\tilde{\varvec{u}}=\frac{\varvec{u}}{U}$. Combining Eqs. (23) and (24), we can rearrange the resulting equation to write $\kappa '$ in terms of $C_{\text {C1}}$ and $C_\mathrm{VC\cdot \mathrm{C1}}$. We can convert $\kappa '$ to $\kappa$ using $\kappa =\kappa '\times 10^{-2} \text {dyn s}\;\text {cm}^{-2}$. Defining the constants $\beta =6.61\times 10^{-17}$ $\hbox {cm}^2$ and $\alpha =-2.70$, we can write $\kappa$ as $\beta [\frac{M_{\text {C1}}(C_{\text {C1}}+C_{VC\cdot C1})}{1\;\text {kg}\;\text {dm}^{-3}}]^{\alpha }$. Substituting this equation for $\kappa$ into Eq. (1) and nondimensionalising the variables, we can write the nondimensionalised Brinkman’s equation as follows,

25 $$\begin{aligned} \tilde{\varvec{\nabla }}\tilde{P}=-\frac{\mu U L}{P^\mathrm{DA}\beta \left( M_{\text {C1}}C_\mathrm{C1,s}\right) ^{\alpha }}\frac{\tilde{\varvec{u}}}{\left( \tilde{C}_{\text {C1}}+\frac{C_\mathrm{VC \cdot C1,s}}{C_\mathrm{C1,s}}\tilde{C}_\mathrm{VC \cdot C1}\right) ^{\alpha }}+\frac{\mu U}{L P^\mathrm{DA}}\tilde{\varvec{\nabla }}^2\tilde{\varvec{u}}. \end{aligned}$$

To simplify Eq. (25), we will lump the parameters into dimensionless groups, $\eta _{1}=\frac{\mu U L}{P^\mathrm{DA}\beta (M_{\text {C1}}C_\mathrm{C1,s})^{\alpha }}$, $\eta _{2}=\frac{C_\mathrm{VC \cdot C1,s}}{C_\mathrm{C1,s}}$, and $\eta _{3}=\frac{\mu U}{L P^\mathrm{DA}}$. To determine the scale of $\varvec{u}$, we can use either $\eta _{1}$ or $\eta _{3}$. Choosing $\eta _{1}=1$ will lead to $U=1.371\times 10^{-4}$ µm/s; choosing $\eta _{3}=1$ will lead to $U=8.891$ m/s. The velocity for an interstitial flow is reported to range from 0.1 to 2 µm/s (Swartz and Fleury 2007), so $\eta _{1}=1$ gives a more appropriate velocity scale. This choice also leads to $\eta _{3}=1.542\times 10^{-11}$. Therefore, Eq. (25) becomes

26 $$\begin{aligned} \tilde{\varvec{\nabla }}\tilde{P}=-\frac{\tilde{\varvec{u}}}{\left( \tilde{C}_{\mathrm{C1}}+\eta _{2}\tilde{C}_{\mathrm{VC} \cdot \mathrm{C1}}\right) ^{\alpha }}+\eta _{3}\tilde{\varvec{\nabla }}^2\tilde{\varvec{u}}. \end{aligned}$$

Nondimensionalising Eq. (2) and the boundary conditions, then defining the dimensionless groups $\eta _\mathrm{DA}=\frac{L_\mathrm{DA}P^\mathrm{DA}}{U}$, $\eta _\mathrm{PCV}=\frac{L_\mathrm{PCV}P^\mathrm{DA}}{U}$, and $\eta _\mathrm{DLAV}=\frac{2L_\mathrm{DLAV}P^\mathrm{DA}}{U}$, we will obtain the remaining nondimensionalised interstitial flow equations and their boundary conditions,

27 $$\begin{aligned} \tilde{\varvec{\nabla }} \cdot \tilde{{\varvec{u}}}= & {} 0, \end{aligned}$$

28 $$\begin{aligned} -\varvec{n} \cdot \tilde{\varvec{u}}= & {} \eta _\mathrm{DA} \left( 1-\tilde{P}\right) \qquad \tilde{\varvec{x}} \in \partial \varOmega _\mathrm{DA}, \end{aligned}$$

29 $$\begin{aligned} -\varvec{n} \cdot \tilde{\varvec{u}}= & {} \eta _\mathrm{PCV} \left( \frac{P^\mathrm{PCV}}{P^\mathrm{DA}}-\tilde{P}\right) \qquad \tilde{\varvec{x}} \in \partial \varOmega _\mathrm{PCV}, \end{aligned}$$

30 $$\begin{aligned} -\varvec{n} \cdot \tilde{\varvec{u}}= & {} \eta _\mathrm{DLAV} \left( \frac{P^\mathrm{DLAV}}{P^\mathrm{DA}}-\tilde{P}\right) \qquad \tilde{\varvec{x}} \in \partial \varOmega _\mathrm{DLAV}, \; \text {and} \end{aligned}$$

31 $$\begin{aligned} \tilde{\varvec{u}}= & {} 0 \qquad \tilde{\varvec{x}} \in \partial \varOmega _{x,y} \; \text {and} \; \partial \varOmega _\mathrm{LEC/IS+}. \end{aligned}$$

Second, we need to nondimensionalise the equations governing the concentration fields in the interstitial space, (7) and (12). To do so, we need to introduce $\tilde{D}^\mathrm{eff}_{i}=\frac{D^\mathrm{eff}_{i}\tau }{L^{2}}$, $\lambda _{7}=\frac{U\tau }{L}$, and $\tilde{R}^\mathrm{IS}_{i}=\frac{R^\mathrm{IS}_{i}\tau }{C_{i,s}}$. Then, the nondimensionalised reaction–diffusion–convection equation takes the form

32 $$\begin{aligned} \frac{\partial \tilde{C}_{i}}{\partial \tilde{t}}=\tilde{\varvec{\nabla }} \cdot \left[ \tilde{D}^\mathrm{eff}_{i}\tilde{\varvec{\nabla }}\left( \frac{\tilde{C}_{i}}{\omega }\right) -\lambda _{7}\tilde{\varvec{u}}\tilde{C}_{i}\right] + \tilde{R}^\mathrm{IS}_{i}. \end{aligned}$$

We will define the dimensionless parameters $\lambda _{5}=v_{{\text {C1h}}}M_{\text {C1}}C_\mathrm{C1,s}$ and $\lambda _{6}=v_{{\text {C1h}}}M_{\text {C1}}C_\mathrm{VC \cdot C1,s}$. Then, we can write $\omega$ as

33 $$\begin{aligned} \omega =1-\lambda _{5}\tilde{C}_{\text {C1}}-\lambda _{6}\tilde{C}_\mathrm{VC \cdot C1}. \end{aligned}$$

$\tilde{D}^\mathrm{eff}_{i}$ can be expressed in terms of the dimensionless parameters $\lambda _{1,i}=\frac{D^{\infty }_{i}\tau }{L^{2}}$, $\lambda _{2,i}=\frac{k_\mathrm{B}T}{6\pi \mu D^{\infty }_{i}r_{f}}$, $\lambda _{3}=v_{\text {C1}}M_{\text {C1}}C_\mathrm{C1,s}$, and $\lambda _{4}=v_{\text {C1}}M_{\text {C1}}C_\mathrm{VC \cdot \mathrm{C1,s}}$. This expression is

34 $$\begin{aligned} \tilde{D}^\mathrm{eff}_{i}=\lambda _{1,i}\exp \left( -\lambda _{2,i}\sqrt{\lambda _{3}\tilde{C}_{\text {C1}}+\lambda _{4}\tilde{C}_\mathrm{VC \cdot C1}}\right) . \end{aligned}$$

For the immobile species in the interstitial space, the reaction–diffusion–convection equation is reduced to

35 $$\begin{aligned} \frac{\partial \tilde{C}_{i}}{\partial \tilde{t}}= \tilde{R}^\mathrm{IS}_{i}. \end{aligned}$$

Third, we will nondimensionalise the equations governing the concentration fields in the LEC domain, (13) and (14). After introducing $\tilde{R}^\mathrm{LEC}_{i}=\frac{R^\mathrm{LEC}_{i}\tau }{C_{i,s}}$, we can write down

36 $$\begin{aligned} \frac{\partial \tilde{C}_{i}}{\partial \tilde{t}}=\lambda _{1,i}\tilde{\varvec{\nabla }}^{2}\tilde{C}_{i}+ \tilde{R}^\mathrm{LEC}_{i}. \end{aligned}$$

For the immobile species in the LEC domain, the reaction–diffusion equation is reduced to

37 $$\begin{aligned} \frac{\partial \tilde{C}_{i}}{\partial \tilde{t}}= \tilde{R}^\mathrm{LEC}_{i}. \end{aligned}$$

Fourth, we will nondimensionalise the boundary and initial conditions of the concentrations. Equations (15–17), which apply to proMMP2, MMP2, TIMP2, MMP2$\cdot$TIMP2, and VEGFC, have the following nondimensionalised forms,

38 $$\begin{aligned} \varvec{n} \cdot \left[ \tilde{D}^\mathrm{eff}_{i}\tilde{\varvec{\nabla }}\left( \frac{\tilde{C}_{i}}{\omega }\right) -\lambda _{7}\tilde{\varvec{u}}\tilde{C}_{i}\right]= & {} 0 \qquad \tilde{\varvec{x}} \in \partial \varOmega _{x,y}, \end{aligned}$$

39 $$\begin{aligned} \varvec{n} \cdot \left[ \tilde{D}^\mathrm{eff}_{i}\tilde{\varvec{\nabla }}\left( \frac{\tilde{C}_{i}}{\omega }\right) -\lambda _{7}\tilde{\varvec{u}}\tilde{C}_{i}\right]= & {} 0 \qquad \tilde{\varvec{x}} \in \partial \varOmega _\mathrm{PCV}, \; \text {and} \end{aligned}$$

40 $$\begin{aligned} \varvec{n} \cdot \left[ \tilde{D}^\mathrm{eff}_{i}\tilde{\varvec{\nabla }}\left( \frac{\tilde{C}_{i}}{\omega }\right) -\lambda _{7}\tilde{\varvec{u}}\tilde{C}_{i}\right]= & {} 0 \qquad \tilde{\varvec{x}} \in \partial \varOmega _\mathrm{DLAV}. \end{aligned}$$

The boundary conditions for proMMP2, MMP2, TIMP2, and MMP2$\cdot$TIMP2 on the DA’s surface, represented by Eq. (18), have the following nondimensionalised form,

41 $$\begin{aligned} \varvec{n} \cdot \left[ \tilde{D}^\mathrm{eff}_{i}\tilde{\varvec{\nabla }}\left( \frac{\tilde{C}_{i}}{\omega }\right) -\lambda _{7}\tilde{\varvec{u}}\tilde{C}_{i}\right] =0 \qquad \tilde{\varvec{x}} \in \partial \varOmega _\mathrm{DA}. \end{aligned}$$

With $\lambda ^\mathrm{DA}_\mathrm{VC}=\frac{R^\mathrm{DA}_\mathrm{VC}\tau }{C_{\mathrm{VC,s}}L}$ being the dimensionless production rate of VEGFC, we can scale $C_\mathrm{VC}$ by setting $\lambda ^\mathrm{DA}_\mathrm{VC}=1$, so $C_\mathrm{VC,s}=1.64\times 10^{-10}$ M. Then, the influx of VEGFC from the DA surface into the interstitial space, Eq. (19), has the nondimensionalised form

42 $$\begin{aligned} {-}\varvec{n} \cdot \left[ -\tilde{D}^\mathrm{eff}_{i}\tilde{\varvec{\nabla }}\left( \frac{\tilde{C}_{i}}{\omega }\right) +\lambda _{7}\tilde{\varvec{u}}\tilde{C}_{i}\right] =\lambda ^\mathrm{DA}_\mathrm{VC} \qquad \tilde{\varvec{x}} \in \partial \varOmega _\mathrm{DA}. \end{aligned}$$

The boundary conditions on the LEC’s surface, Eqs. (20–22), are then nondimensionalised to give

43 $$\begin{aligned} \varvec{n} \cdot \left[ \tilde{D}^\mathrm{eff}_{i}\tilde{\varvec{\nabla }}\left( \frac{\tilde{C}_{i}}{\omega }\right) -\lambda _{7}\tilde{\varvec{u}}\tilde{C}_{i}\right] \Big \arrowvert _{\partial \varOmega _\mathrm{LEC/IS+}}= & {} -\varvec{n} \cdot \left( \lambda _{1,i}\tilde{\varvec{\nabla }}\tilde{C}_{i}\right) \Big \arrowvert _{\partial \varOmega _\mathrm{LEC/IS-}}, \end{aligned}$$

44 $$\begin{aligned} \tilde{C}_{i}\arrowvert _{\partial \varOmega _\mathrm{LEC/IS+}}= & {} \tilde{C}_{i}\arrowvert _{\partial \varOmega _\mathrm{LEC/IS-}}, \; \text {and} \end{aligned}$$

45 $$\begin{aligned} \varvec{n} \cdot \left[ \tilde{D}^\mathrm{eff}_{i}\tilde{\varvec{\nabla }}\left( \frac{\tilde{C}_{i}}{\omega }\right) -\lambda _{7}\tilde{\varvec{u}}\tilde{C}_{i}\right]= & {} 0 \qquad \tilde{\varvec{x}} \in \partial \varOmega _\mathrm{LEC/IS+}. \end{aligned}$$

The continuity condition defined by Eqs. (43) and (44) applies to proMMP2, MMP2, and TIMP2. The no-flux condition defined by Eq. (45) applies to MMP2$\cdot$TIMP2 and VEGFC.

The initial concentrations of MT1-MMP in the LEC domain and collagen I in the interstitial space domain are $\tilde{C}_\mathrm{MT1}=1$ and $\tilde{C}_{\text {C1}}=1$, respectively.

Finally, we need to define the nondimensionalised reaction terms, $\tilde{R}^\mathrm{IS}_{i}=\frac{R^\mathrm{IS}_{i}\tau }{C_{i,s}}$ and $\tilde{R}^\mathrm{LEC}_{i}=\frac{R^\mathrm{LEC}_{i}\tau }{C_{i,s}}$, in terms of nondimensionalised concentrations. We need the remaining concentration scales. We will scale $C_{\text {M2P}}$ by setting the dimensionless production term of proMMP2 to unity, $\frac{P_{\text {M2P}}\tau }{C_\mathrm{M2P,s}}=1$. This leads to $C_\mathrm{M2P,s}=1.14\times 10^{-3}$ M. We will set the dimensionless production term of MMP2 to unity, $\frac{k^\mathrm{eff}_\mathrm{act}\tau C_\mathrm{MT1\cdot T2\cdot M2P,s}C_\mathrm{MT1,s}}{C_\mathrm{M2,s}}=1$. This gives the concentration scale for MMP2, $C_\mathrm{M2,s}=3.94\times 10^{-5}$ M. Setting the dimensionless production term of TIMP2 to unity, $\frac{P_{\text {T2}}\tau }{C_\mathrm{T2,s}}=1$, we can obtain the concentration scale $C_\mathrm{T2,s}=6.65\times 10^{-6}$ M. If the binding term of MMP2 and TIMP2 is set to unity, $\frac{k^\mathrm{on}_{\text {M2,T2}}\tau C_\mathrm{M2,s}C_\mathrm{T2,s}}{C_{{\text {M2}}\cdot {\text {T2,s}}}}=1$, the concentration scale is $C_{{\text {M2}}\cdot {\text {T2,s}}}=6.68\times 10^{1}$ M. Similarly, by equating the binding term of VEGFC and collagen I to unity, $\frac{k^\mathrm{on}_{\mathrm{VC,C1}}\tau C_{\mathrm{VC,s}}C_\mathrm{C1,s}}{C_\mathrm{VC\cdot C1,s}}=1$, we can scale the concentration of bound VEGFC, $C_\mathrm{VC \cdot C1,s}=8.93\times 10^{-5}$ M. With all the scales determined, the reaction terms can easily be worked out. They are summarised in Table 10.

**Table 10:** Nondimensionalised reaction terms in the interstitial space and lymphatic endothelial cell domains

Reaction term	Form	Equations
$\tilde{R}^\mathrm{IS}_{\text {M2P}}$	$-\lambda _{11} \tilde{C}_{\text {M2P}}$	(32)
$\tilde{R}^\mathrm{IS}_{\text {M2}}$	$-\lambda _{13}\tilde{C}_{\text {M2}}\tilde{C}_{\text {T2}}+\lambda _{14}\tilde{C}_{{\text {M2}}\cdot {\text {T2}}}-\lambda _{15} \tilde{C}_{\text {M2}}$	(32)
$\tilde{R}^\mathrm{IS}_{\text {T2}}$	$-\lambda _{19}\tilde{C}_{\text {M2}}\tilde{C}_{\text {T2}}+\lambda _{20}\tilde{C}_{{\text {M2}}\cdot {\text {T2}}}-\lambda _{21}\tilde{C}_{\text {T2}}$	(32)
$\tilde{R}^\mathrm{IS}_{{\text {M2}}\cdot {\text {T2}}}$	$\tilde{C}_{\text {M2}}\tilde{C}_{\text {T2}}-\lambda _{23} \tilde{C}_{{\text {M2}}\cdot {\text {T2}}}-\lambda _{24} \tilde{C}_{{\text {M2}}\cdot {\text {T2}}}$	(32)
$\tilde{R}^\mathrm{IS}_\mathrm{VC}$	$-\lambda _{25}\tilde{C}_\mathrm{VC}\tilde{C}_{\text {C1}}+\lambda _{26}\tilde{C}_\mathrm{VC\cdot C1}-\lambda _{27}\tilde{C}_\mathrm{VC}$	(32)
$\tilde{R}^\mathrm{IS}_{\text {C1}}$	$\frac{-\lambda _{28}\tilde{C}_{\text {M2}}\tilde{C}_{\text {C1}}}{K^\mathrm{M2,C1}_{M}+C_\mathrm{C1,s}\tilde{C}_{\text {C1}}}-\lambda _{29}\tilde{C}_\mathrm{VC}\tilde{C}_{\text {C1}}+\lambda _{30}\tilde{C}_\mathrm{VC\cdot C1}$	(35)
$\tilde{R}^\mathrm{IS}_\mathrm{VC\cdot C1}$	$\tilde{C}_\mathrm{VC}\tilde{C}_{\text {C1}}-\lambda _{32} \tilde{C}_\mathrm{VC\cdot C1}$	(35)
$\tilde{R}^\mathrm{LEC}_{\text {M2P}}$	$1-\lambda _{9}\tilde{C}_\mathrm{MT1\cdot T2}\tilde{C}_{\text {M2P}}+\lambda _{10}\tilde{C}_\mathrm{MT1\cdot T2\cdot M2P}$	(36)
$\tilde{R}^\mathrm{LEC}_{\text {M2}}$	$\tilde{C}_\mathrm{MT1\cdot T2\cdot M2P}\tilde{C}_\mathrm{MT1}$	(36)
$\tilde{R}^\mathrm{LEC}_{\text {T2}}$	$1-\lambda _{17}\tilde{C}_\mathrm{MT1}\tilde{C}_{\text {T2}}+\lambda _{18}\tilde{C}_\mathrm{MT1\cdot T2}$	(36)
$\tilde{R}^\mathrm{LEC}_\mathrm{MT1}$	$-\lambda _{33}\tilde{C}_\mathrm{MT1}\tilde{C}_{\text {T2}}+\lambda _{34}\tilde{C}_\mathrm{MT1\cdot T2}$	(37)
$\tilde{R}^\mathrm{LEC}_\mathrm{MT1 \cdot T2}$	$\lambda _{35}\tilde{C}_\mathrm{MT1}\tilde{C}_{\text {T2}}-\lambda _{36}\tilde{C}_\mathrm{MT1\cdot T2}$	(37)
	$$-\lambda _{37}\tilde{C}_\mathrm{MT1\cdot T2}\tilde{C}_{\text {M2P}}+\lambda _{38}\tilde{C}_\mathrm{MT1\cdot T2\cdot M2P}$$	
	$$+\lambda _{39}\tilde{C}_\mathrm{MT1\cdot T2\cdot M2P}\tilde{C}_\mathrm{MT1}$$	
$\tilde{R}^\mathrm{LEC}_\mathrm{MT1 \cdot T2 \cdot M2P}$	$\lambda _{40}\tilde{C}_\mathrm{MT1\cdot T2}\tilde{C}_{\text {M2P}}-\lambda _{41}\tilde{C}_\mathrm{MT1\cdot T2\cdot M2P}$	(37)
	$$-\lambda _{42}\tilde{C}_\mathrm{MT1\cdot T2\cdot M2P}\tilde{C}_\mathrm{MT1}$$	

M2P, proMMP2; M2, MMP2; T2, TIMP2; VC, VEGFC; C1, collagen 1; MT1, MT1-MMP

*IS* interstitial space, *LEC* lymphatic endothelial cell

### Simplification

After nondimensionalisation, we can spot several opportunities for simplifying the model.

First, the nonlinear term in Eq. (26), $\frac{\tilde{\varvec{u}}}{(\tilde{C}_{\text {C1}}+\eta _{2}\tilde{C}_{\mathrm{VC} \cdot \mathrm{C1}})^{\alpha }}$, can be linearised if $\tilde{C}_{\text {C1}}$ and $\tilde{C}_{\mathrm{VC} \cdot \mathrm{C1}}$ differ by orders of magnitude. After factorisation, the nonlinear term becomes $\tilde{\varvec{u}}\tilde{C}_{\text {C1}}^{-\alpha }(1+\frac{\eta _{2}\tilde{C}_\mathrm{VC \cdot C1}}{\tilde{C}_{\text {C1}}})^{-\alpha }$, which can be expanded as a Taylor series at $\frac{\eta _{2}\tilde{C}_\mathrm{VC \cdot C1}}{\tilde{C}_{\text {C1}}} \approx 0$. The result is

46 $$\begin{aligned}&\tilde{\varvec{u}}\tilde{C}_{\text {C1}}^{-\alpha } \left[ 1-\alpha \frac{\eta _{2}\tilde{C}_\mathrm{VC \cdot C1}}{\tilde{C}_{\text {C1}}}+\frac{\alpha (\alpha +1)}{2}\left( \frac{\eta _{2}\tilde{C}_\mathrm{VC \cdot C1}}{\tilde{C}_{\text {C1}}}\right) ^{2}\right. \nonumber \\&\quad \left. -\,\frac{\alpha \left( \alpha +1\right) \left( \alpha +2\right) }{6}\left( \frac{\eta _{2}\tilde{C}_\mathrm{VC \cdot C1}}{\tilde{C}_{\text {C1}}}\right) ^{3}+\cdots \right] . \end{aligned}$$

This expansion assumes that $\left| \frac{\eta _{2}\tilde{C}_\mathrm{VC \cdot C1}}{\tilde{C}_{\text {C1}}}\right| \ll 1$ is true in the interstitial space domain. The binding rate constant of VEGFC and collagen I in $\tilde{R}^\mathrm{IS}_\mathrm{VC\cdot C1}$ is $\lambda _{31}=1$; the unbinding rate constant of the same process is $\lambda _{32}=1.56\times 10^{2}$. The initial concentration of collagen I is unity, but it is zero for VEGFC bound by collagen I. It follows that $\tilde{C}_\mathrm{VC \cdot C1}$ must be orders of magnitude smaller than $\tilde{C}_{\text {C1}}$ all the time. We also know that $\eta _{2}=0.255$. Taken together, the required assumption is a reasonable one to make. By the same argument, the terms beyond unity in the square brackets can be neglected, leading to a simplified version of Eq. (26),

47 $$\begin{aligned} \tilde{\varvec{\nabla }}\tilde{P}=-\tilde{\varvec{u}}\tilde{C}_{\text {C1}}^{-\alpha }+\eta _{3}\tilde{\varvec{\nabla }}^2\tilde{\varvec{u}}. \end{aligned}$$

To make it easier to solve the model numerically, we will divide Eq. (47) by $\eta _{3}$ and define the simulated pressure, $\tilde{P^{s}}=\frac{\tilde{P}}{\eta _{3}}$, resulting in

48 $$\begin{aligned} \tilde{\varvec{\nabla }}\tilde{P^{s}}=-\frac{\tilde{\varvec{u}}\tilde{C}_{\text {C1}}^{-\alpha }}{\eta _{3}}+\tilde{\varvec{\nabla }}^2\tilde{\varvec{u}}. \end{aligned}$$

Second, the blood vessels are very leaky. In the equations representing the transvascular fluxes of interstitial fluid, (28–30), the dimensionless vascular permeabilities, $\eta _\mathrm{DA}=1.317\times 10^{14}$, $\eta _\mathrm{PCV}=1.317\times 10^{14}$, and $\eta _\mathrm{DLAV}=2.634\times 10^{14}$, are all orders of magnitude larger than unity. In physiological terms, the blood vessels are so leaky that we can ignore any transvascular pressure drops. As a result, the fluxes can be replaced by constant pressures, leading to

49 $$\begin{aligned} \tilde{P^{s}}=\frac{1}{\eta _{3}} \qquad \tilde{\varvec{x}} \in \partial \varOmega _\mathrm{DA}, \end{aligned}$$

50 $$\begin{aligned} \tilde{P^{s}}=0 \qquad \tilde{\varvec{x}} \in \partial \varOmega _\mathrm{PCV}, \; \text {and} \end{aligned}$$

51 $$\begin{aligned} \tilde{P^{s}}=0 \qquad \tilde{\varvec{x}} \in \partial \varOmega _\mathrm{DLAV}. \end{aligned}$$

Third, we can linearise the diffusive term in Eq. (32), $\tilde{D}^\mathrm{eff}_{i}\tilde{\varvec{\nabla }}(\frac{\tilde{C}_{i}}{\omega })$. We will start with $\frac{1}{\omega }=\left( 1-\lambda _{5}\tilde{C}_{\text {C1}}-\lambda _{6}\tilde{C}_\mathrm{VC \cdot C1}\right) ^{-1}$. We can apply $(1+x)^{-1} \approx 1-x+x^{2}-x^{3}+x^{4}-\cdots$ around $x=0$ (Abramowitz and Stegun 1964) to obtain

52 $$\begin{aligned}&\left[ 1+\left( -\lambda _{5}\tilde{C}_{\text {C1}}-\lambda _{6}\tilde{C}_\mathrm{VC \cdot C1}\right) \right] ^{-1}\nonumber \\&\quad =1-\left( -\lambda _{5}\tilde{C}_{\text {C1}}-\lambda _{6}\tilde{C}_\mathrm{VC \cdot C1}\right) +\left( -\lambda _{5}\tilde{C}_{\text {C1}}-\lambda _{6}\tilde{C}_\mathrm{VC \cdot C1}\right) ^2 + \cdots \end{aligned}$$

This approximation assumes that $\left| \lambda _{5}\tilde{C}_{\text {C1}}+\lambda _{6}\tilde{C}_\mathrm{VC \cdot C1}\right| \ll 1$ holds in the interstitial space. Since $\lambda _{5}=1.98\times 10^{-1}$ and $\tilde{C}_{\text {C1}}\le 1$ are true, $\lambda _{5}\tilde{C}_{\text {C1}} \ll 1$ must hold too. With respect to the second term, we know that $\lambda _{6}=5.06\times 10^{-2}$ and $\tilde{C}_\mathrm{VC \cdot C1} \ll \tilde{C}_{\text {C1}}$, so it is orders of magnitude less than unity. The assumption is a valid one. Also, the terms beyond $\lambda _{5}\tilde{C}_{\text {C1}}$ are negligible. The result is $\frac{1}{\omega } \approx 1+\lambda _{5}\tilde{C}_{\text {C1}}$.

The effective diffusivity can be simplified along similar lines. The Taylor series expansion of a general exponential function around $x=0$ is $e^{x} \approx 1+x+\frac{x^{2}}{2!}+\frac{x^{3}}{3!}+\cdots$ We can apply it to the exponential term in $\tilde{D}^\mathrm{eff}_{i}=\lambda _{1,i}\exp (-\lambda _{2,i}\sqrt{\lambda _{3}\tilde{C}_{\text {C1}}+\lambda _{4}\tilde{C}_\mathrm{VC \cdot C1}})$, resulting in

53 $$\begin{aligned}&\exp \left( -\lambda _{2,i}\sqrt{\lambda _{3}\tilde{C}_{\text {C1}}+\lambda _{4}\tilde{C}_\mathrm{VC \cdot C1}}\right) =1-\lambda _{2,i}\sqrt{\lambda _{3}\tilde{C}_{\text {C1}}+\lambda _{4}\tilde{C}_\mathrm{VC \cdot C1}}\nonumber \\&\quad +\,\frac{\lambda _{2,i}^{2}\left( \lambda _{3}\tilde{C}_{\text {C1}}+\lambda _{4}\tilde{C}_\mathrm{VC \cdot C1}\right) }{2!}+\cdots \end{aligned}$$

As usual, we need the magnitude of the exponent to be much smaller than one for Taylor expansion to work. Since $\lambda _{3}=7.88\times 10^{-2}$ and $\tilde{C}_{\text {C1}}\le 1$ are true, the first term in the square root is much smaller than one; $\lambda _{4}=2.01\times 10^{-2}$ and $\tilde{C}_\mathrm{VC \cdot C1} \ll \tilde{C}_{\text {C1}}$ mean the same for the second term. The square root $\sqrt{\lambda _{3}\tilde{C}_{\text {C1}}+\lambda _{4}\tilde{C}_\mathrm{VC \cdot C1}}$ is on the order of $1\times 10^{-1}$ or smaller. Since $\lambda _{2,i}$ is on the order of unity, this expansion is justified. For the same reason, the terms beyond $-\lambda _{2,i}\sqrt{\lambda _{3}\tilde{C}_{\text {C1}}+\lambda _{4}\tilde{C}_\mathrm{VC \cdot C1}}$ in the expansion are negligible, thus reducing the diffusivity to $\lambda _{1,i}\left( 1-\lambda _{2,i}\sqrt{\lambda _{3}\tilde{C}_{\text {C1}}+\lambda _{4}\tilde{C}_\mathrm{VC \cdot C1}}\right)$.

The square root in $\lambda _{1,i}\left( 1-\lambda _{2,i}\sqrt{\lambda _{3}\tilde{C}_{\text {C1}}+\lambda _{4}\tilde{C}_\mathrm{VC \cdot C1}}\right)$ can be simplified even further. Rewriting it as $\sqrt{\lambda _{3}\tilde{C}_{\text {C1}}}(1+\frac{\lambda _{4}\tilde{C}_\mathrm{VC \cdot C1}}{\lambda _{3}\tilde{C}_{\text {C1}}})^{1/2}$ and knowing that $\left| \frac{\lambda _{4}\tilde{C}_\mathrm{VC \cdot C1}}{\lambda _{3}\tilde{C}_{\text {C1}}}\right| \ll 1$, we will expand it as a Taylor series around $\frac{\lambda _{4}\tilde{C}_\mathrm{VC \cdot C1}}{\lambda _{3}\tilde{C}_{\text {C1}}}=0$,

54 $$\begin{aligned} \sqrt{\lambda _{3}\tilde{C}_{\text {C1}}}\left[ 1+\frac{1}{2}\left( \frac{\lambda _{4}\tilde{C}_\mathrm{VC \cdot C1}}{\lambda _{3}\tilde{C}_{\text {C1}}}\right) -\frac{1}{8}\left( \frac{\lambda _{4}\tilde{C}_\mathrm{VC \cdot C1}}{\lambda _{3}\tilde{C}_{\text {C1}}}\right) ^{2} + \cdots \right] \end{aligned}$$

Because the terms beyond unity are negligible, the effective diffusivity is simplified to $\lambda _{1,i}\left( 1-\lambda _{2,i}\sqrt{\lambda _{3}\tilde{C}_{\text {C1}}}\right)$.

The simplified Eq. (32) is

55 $$\begin{aligned} \frac{\partial \tilde{C}_{i}}{\partial \tilde{t}}=\tilde{\varvec{\nabla }} \cdot \left[ \lambda _{1,i}\left( 1-\lambda _{2,i}\sqrt{\lambda _{3}\tilde{C}_{\text {C1}}}\right) \tilde{\varvec{\nabla }}\left( \tilde{C}_{i}+\lambda _{5}\tilde{C}_{\text {C1}}\tilde{C}_{i}\right) -\lambda _{7}\varvec{\tilde{u}}\tilde{C}_{i}\right] +\tilde{R}_{i}. \end{aligned}$$

The simplified expressions $\frac{1}{\omega } \approx 1+\lambda _{5}\tilde{C}_{\text {C1}}$ and $\tilde{D}^\mathrm{eff}_{i}=\lambda _{1,i}\left( 1-\lambda _{2,i}\sqrt{\lambda _{3}\tilde{C}_{\text {C1}}}\right)$ will also be applied to the boundary conditions.

## Numerical Experiments

In this section, we will solve the mathematical model numerically, including numerical experiments which solve the model with modified model structures and parameters. We will start by explaining how we will solve the model using COMSOL Multiphysics, including convergence studies to determine the appropriate mesh and time step sizes. Then, we will investigate the spatiotemporal dynamics of VEGFC, MMP2, and collagen I under different circumstances.

### COMSOL Multiphysics Settings

COMSOL Multiphysics version 5.2 is a software package which uses the finite element method to solve partial differential equations numerically. First, the interstitial flow equations, (27) and (48), will be solved alone for the initial velocity field. Second, we will solve the time-dependent equations (32) and (35–37) together with Eqs. (27) and (48).

We will adopt a fully coupled approach to solve the model. It means that Eqs. (27) and (48) will be solved together in the first step, while Eqs. (27), (48), (32), and (35–37) will be solved together in the second step. Since our equations are all nonlinear, discretisation will result in a system of nonlinear algebraic equations. In the first step, we will use an ‘automatic highly nonlinear (Newton)’ solver; the second step will be tackled by the ‘constant (Newton)’ solver. The former has a minimum damping factor of $1\times 10^{-8}$; its damping factor cannot change more than tenfold in one iteration; it terminates when the estimated relative error is less than 0.001. The latter has a constant damping factor of 0.9 and terminates when the estimated relative error is less than 0.01.

The second step runs from $\tilde{t}=0$ to $\tilde{t}=1$. We will use the BDF (backward differentiation formula) method to determine the time steps adaptively. At each time point, the solutions at the previous one or two time steps are used to estimate the time derivatives at the next time step, while the stability of the derivatives determines the step size. As a starting point, we will set a maximum step size of 0.02. We will optimise this criterion after optimising our mesh setting. The mobile species are absent from the geometry initially. They need time to permeate it, so their concentrations will change drastically in the first few time steps. These time steps require tight control. Based on $U=1.371\times 10^{-4}$ µm/s, it takes a particle $7.29\times 10^{3}$ s to transverse 1 $\upmu \hbox {m}$ by convection. From $D^{\infty }_{\text {T2}}=1.10\times 10^{-6}$ $\hbox {cm}^2/\hbox {s}$, the highest diffusivity in our model, we know that the equivalent time is $9.09\times 10^{-3}$ s for diffusion. Nondimensionalising the shorter travelling time, $9.09\times 10^{-3}$ s becomes $2.10\times 10^{-7}$. We will therefore set the initial time step at $1\times 10^{-7}$.

**Fig. 4:**
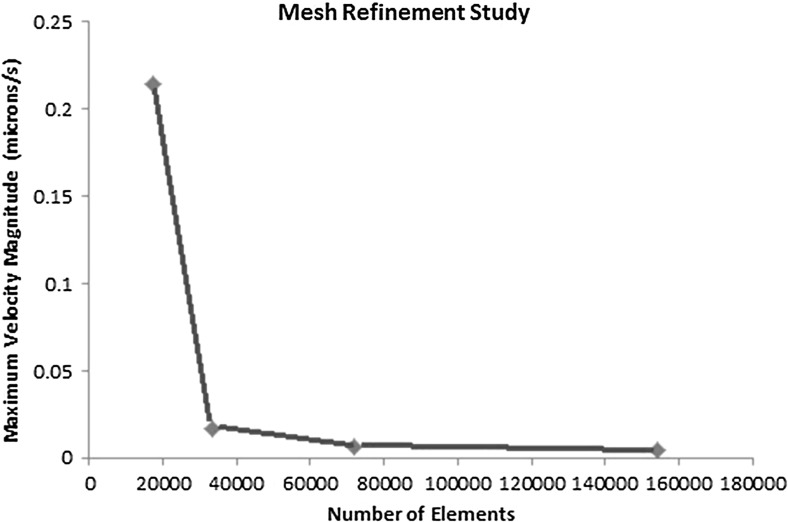
(Color figure online) Convergence plot for the mesh refinement study. It shows how the maximum velocity magnitude at $\tilde{t}=1$ changes with the number of mesh elements. The data points are the numerical results obtained with, from *left* to *right*, the ‘fine’, ‘finer’, ‘extra fine’, and ‘extremely fine’ mesh settings in COMSOL Multiphysics version 5.2

We will perform a convergence study to determine the appropriate mesh size. In COMSOL Multiphysics version 5.2, there are several predefined mesh settings. We will solve the mathematical model with the ‘fine’, ‘finer’, ‘extra fine’, and ‘extremely fine’ mesh settings. We will run the simulations on a desktop computer with an Intel(R) Core(TM) i5-3570 CPU at 3.40 GHz and 16 GB of RAM. Figure 4 is the resulting convergence plot. The interstitial flow ranges from 0.1 to 2 µm/s in speed (Swartz and Fleury 2007). The second data point in Fig. 4 is 0.0173 µm/s, so convergence is achieved after one refinement. Thus, the ‘finer’ mesh setting is sufficient to capture the relevant biological information of the model. With this setting, there are 33562 elements ranging from $1.98\times 10^{-5}$ to 0.037 in nondimensionalised length.

We will next optimise the maximum size of a time step. The equations governing $\varvec{\tilde{u}}$, (27) and (48), are static. A variable controlled by Eq. (35), an ordinary differential equation in time, is more suitable for this convergence study. When the maximum time step is 0.02 and the ‘finer’ mesh setting is used, the minimum $\tilde{C}_{\text {C1}}$ at $\tilde{t}=1$ is 0.9991. When the maximum time step is halved to 0.01, the minimum $\tilde{C}_{\text {C1}}$ at $\tilde{t}=1$ is 0.99909. The difference is 0.001%. The biologically relevant range of $\tilde{C}_{\text {C1}}$ is from 0.01 to 1 (Edds Jr 1958; Levick 1987; Karagiannis and Popel 2006). We can safely assume that a maximum time step of 0.02 is appropriate.

Our geometry is symmetric in the lateral direction. The line of symmetry runs vertically through the whole geometry and passes the centres of the blood vessels and the LEC. We will simulate half of the geometry only to save computational cost. To do so in COMSOL Multiphysics version 5.2, we will remove everything to the right of the line of symmetry from the geometry. Then, we will set the normal component of the velocity and those of the molecular fluxes to zero at the symmetry boundary.

All the simulations discussed in this paper were carried out on the aforementioned desktop computer. With the settings described in this subsection, it took 1300 s to solve the mathematical model. Henceforth, we will call this solution the primary simulation. The other simulations involved similar computational costs.

### Diffusion and Sequestration Act Together

This subsection considers the primary simulation. First, we will determine the dominant transport phenomenon. Péclet number, *Pe*, measures the relative importance of convection and diffusion in a transport process. For our mathematical model, it is $\frac{UL}{D^{\infty }_{i}}=\frac{\lambda _{7}}{\lambda _{1,i}}$. This expression is based on the characteristic velocity scale, but the velocity magnitude varies in space. A better measure is $|\tilde{\varvec{u}}|Pe$, the maximum of which is 0.14909 at $\tilde{t}=1$ for VEGFC. Of all the diffusible species in our model, VEGFC has the smallest $\lambda _{1,i}$, so $|\tilde{\varvec{u}}|Pe$ can only be smaller for another species. Diffusion is the dominant mode of transport in the primary simulation.

**Fig. 5:**
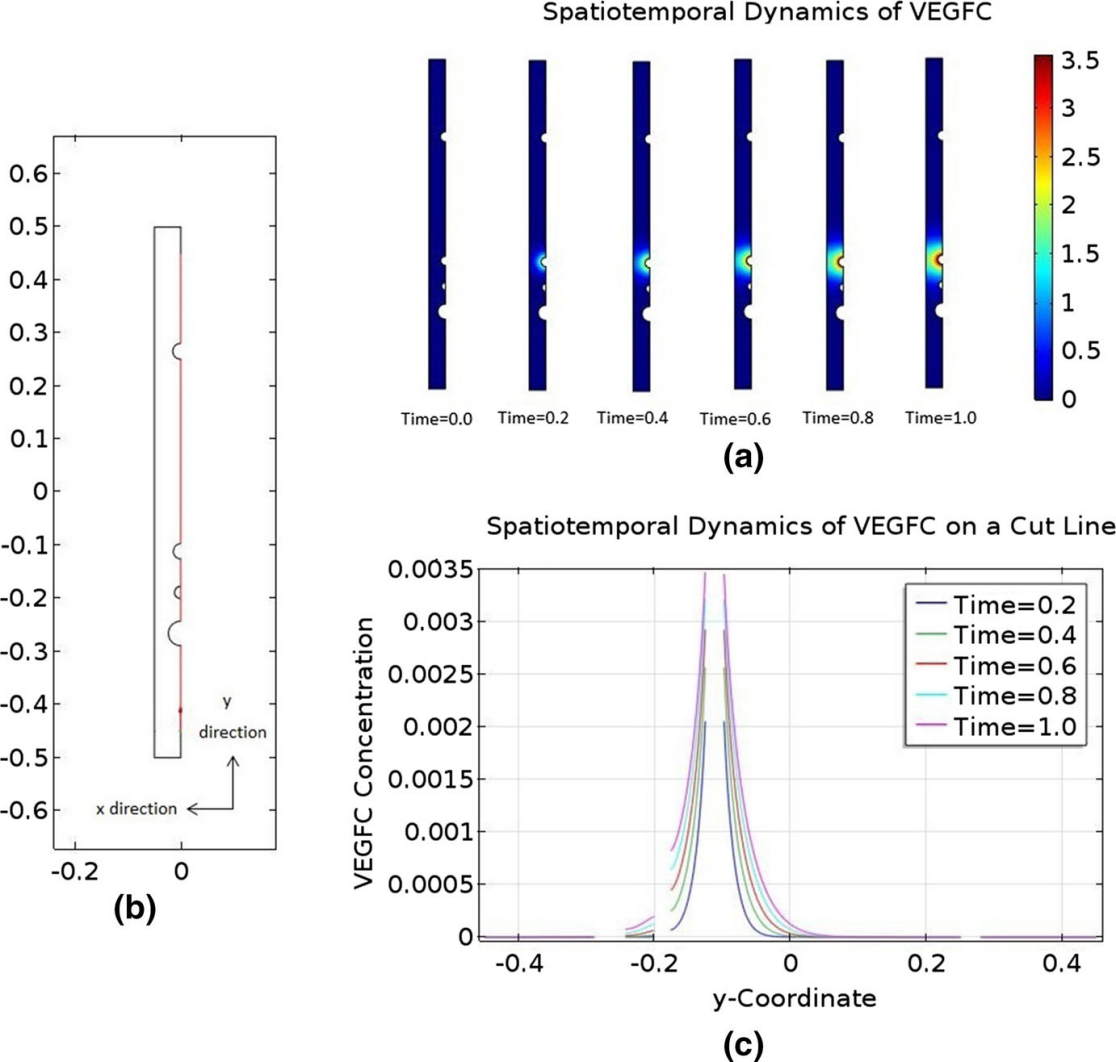
(Color figure online) Spatiotemporal dynamics of VEGFC in the primary simulation. **a** shows the full concentration profiles of VEGFC at different time points. **b** Defines the coordinate system of the geometry and a cut line in the y-direction. The cut line, which is in *red*, goes from $\tilde{y}=-0.45$ to $\tilde{y}=0.45$ at $\tilde{x}=0$. **c** The concentration profiles of VEGFC on this cut line at different time points. The gaps are, from *left* to *right*, the posterior cardinal vein, the lymphatic endothelial cell, the dorsal aorta, and the dorsal longitudinal anastomotic vessel

The spatiotemporal dynamics of $\tilde{C}_\mathrm{VC}$ are shown in Fig. 5. Throughout our time frame of interest, $\tilde{C}_\mathrm{VC}$ peaks at the DA and decreases away from it. This symmetric distribution around the source is consistent with diffusion being the dominant mode of transport. As time passes, VEGFC achieves a wider reach by diffusion. It means that the transport of VEGFC does not equilibrate on this time scale. Because VEGFC is constantly produced, the baseline concentration of it increases with time.

Based on the simulation results, VEGFC is unlikely to be a chemotactic factor because it increases from the PCV to the DA and then decreases to the horizontal myoseptum. If VEGFC is a chemoattractant, it can guide the LEC to the DA, but the cell will remain there instead of moving further to the horizontal myoseptum. If it is a chemorepellent, the LEC should not migrate dorsally at all.

**Fig. 6:**
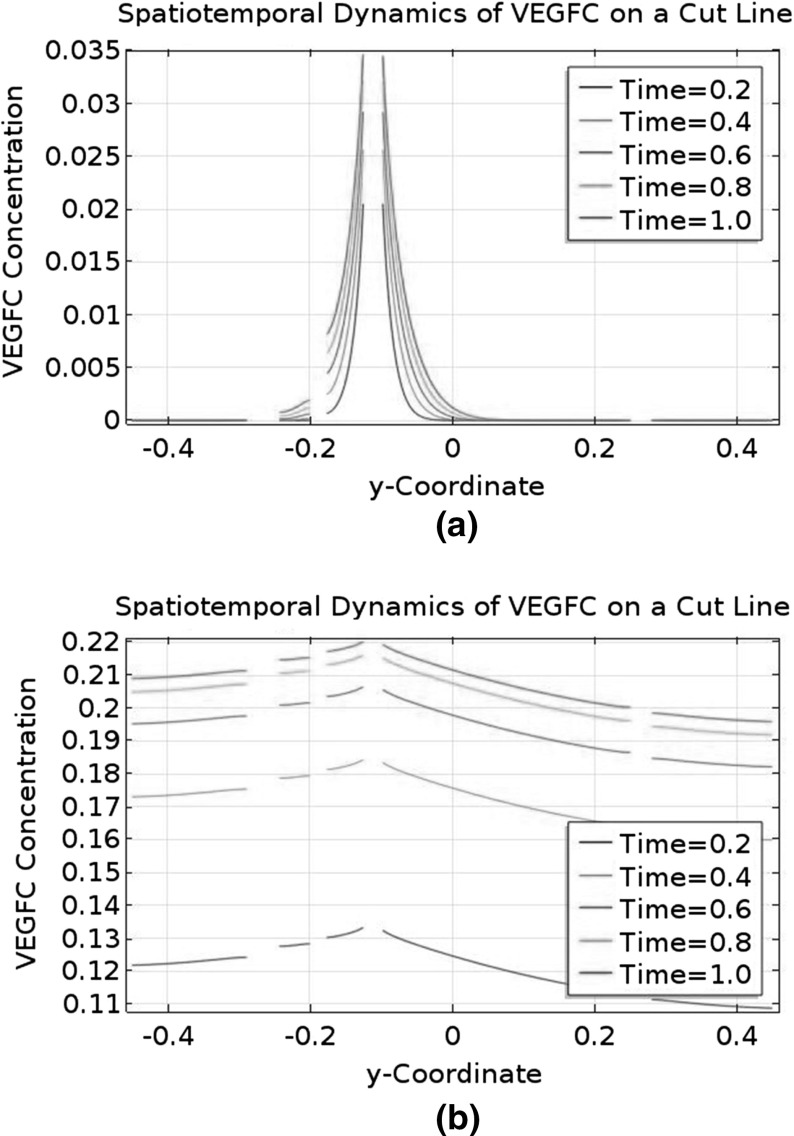
(Color figure online) Spatiotemporal dynamics of VEGFC in two numerical experiments. Both sets of results are shown on the cut line running from $\tilde{y}=-0.45$ to $\tilde{y}=0.45$ at $\tilde{x}=0$. **a** The simulated dynamics when the production rate of VEGFC is increased tenfold. **b** The dynamics simulated without VEGFC–collagen I interactions

On the other hand, we argue that VEGFC is a morphogen for the LEC. The LEC is in a VEGFC gradient which increases towards the DA for the entirety of the simulation. At $\tilde{t}=1$, $\tilde{C}_\mathrm{VC}$ rises from around 0.0001 at the dorsal end of the PCV to 0.0034 at the ventral end of the DA, roughly a thirtyfold increase over 25 $\upmu \hbox {m}$. It is estimated in Gurdon and Bourillot (2001) that a cell can read a threefold change in a morphogen’s concentration over 30 $\upmu \hbox {m}$. This estimate is based on the responses of genes to Dpp and activin gradients. The concentration profile of VEGFC in the primary simulation therefore allows it to be a morphogen. On the other hand, the viable range of known morphogens is from $1\times 10^{-9}$ to $1\times 10^{-11}$ M (Gurdon and Bourillot 2001). $\tilde{C}_\mathrm{VC}$ in the primary simulation is on the order of $1 \times 10^{-13}$ M. Assuming sequestered VEGFC cannot function like free VEGFC, there is insufficient VEGFC. However, our estimated VEGFC production rate and VEGFC–collagen I binding rate constants are crude. If we increase $\lambda ^\mathrm{DA}_\mathrm{VC}$ tenfold in a numerical experiment, the baseline of $\tilde{C}_\mathrm{VC}$ will increase tenfold, but its profile’s shape will remain unchanged. This is illustrated in Fig. 6a.

We will perform another numerical experiment by setting $\lambda _{25}=0$, $\lambda _{26}=0$, $\lambda _{29}=0$, $\lambda _{30}=0$, $\lambda _{31}=0$, and $\lambda _{32}=0$. Biophysically, this means we will turn off any interactions between VEGFC and collagen I. The simulated dynamics in this numerical experiment are shown in Fig. 6b. Although the peak of $\tilde{C}_\mathrm{VC}$ remains at the DA and its baseline still increases with time, the gradients are now too flat for VEGFC to be a morphogen. Sequestration by the ECM in a zebrafish embryo is necessary for VEGFC to function as a morphogen when the embryo is diffusion-dominant.

To conclude, in a diffusion-dominant zebrafish embryo, VEGFC may act as a morphogen, but it is not a chemotactic factor. For it to be a morphogen, it must bind to the ECM to steepen its concentration gradients. Sufficient VEGFC must be produced too because its production rate controls its concentration baseline.

### MMP2 Acts Globally

**Fig. 7:**
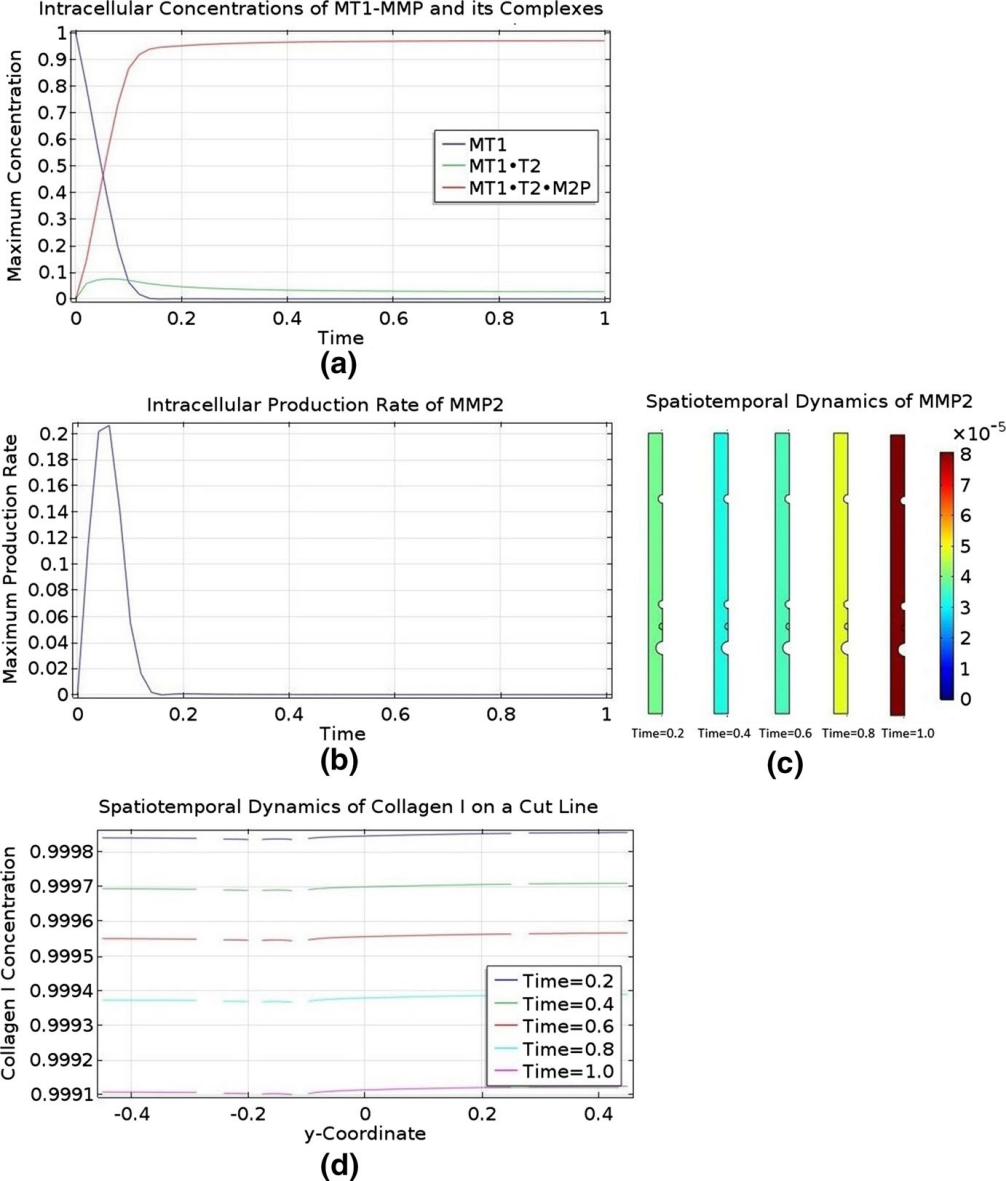
(Color figure online) Behaviour of MMP2 in the primary simulation. **a** The temporal dynamics of MT1-MMP and its complexes inside the lymphatic endothelial cell. The maximum concentration of each species in the cell is plotted at each time point. **b** The production rate of MMP2 in the cell, $\tilde{C}_\mathrm{MT1\cdot T2\cdot M2P}\tilde{C}_\mathrm{MT1}$. The maximum production rate in the cell is plotted at each time point. **c** The concentration profiles of MMP2 at selected time points. **d** The spatiotemporal dynamics of collagen I on the cut line running from $\tilde{y}=-0.45$ to $\tilde{y}=0.45$ at $\tilde{x}=0$. MT1, MT1-MMP; T2, TIMP2; M2P, proMMP2

We will now return to the primary simulation. Figure 7 summarises the simulated behaviour of MMP2. As shown in Fig. 7a, MT1-MMP gets depleted very quickly in the LEC. This rapid depletion of MT1-MMP means there is an initial burst of MMP2 production followed by a prolonged period of low production; MMP2 activation requires MT1-MMP to break up MT1-MMP$\cdot$TIMP2$\cdot$proMMP2. The dynamics of MMP2 production are illustrated by Fig. 7b. From Fig. 7c, we can see that MMP2 is almost homogeneously distributed. Therefore, we can consider its diffusion to be in equilibrium on this time scale. Since MMP2 degrades collagen I, the latter has a nearly homogeneous distribution too. This can be seen in Fig. 7d. The extent of this degradation is less than 0.1% and therefore negligible.

**Fig. 8:**
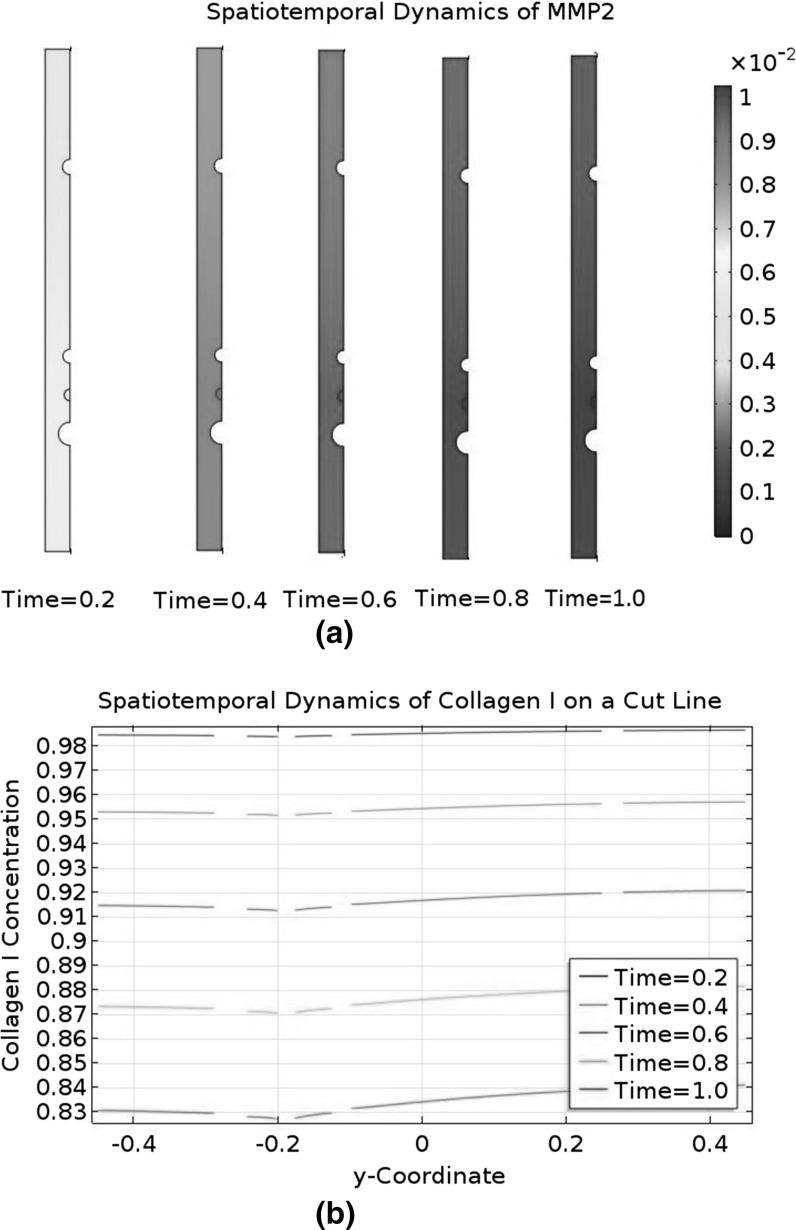
(Color figure online) Behaviour of MMP2 in a numerical experiment which replaces the activation mechanism of MMP2 with a constant production rate. **a** The concentration profiles of MMP2 at selected time points. **b** The spatiotemporal dynamics of collagen I on the cut line running from $\tilde{y}=-0.45$ to $\tilde{y}=0.45$ at $\tilde{x}=0$

However, we know that the production rates of MT1-MMP and TIMP2 are dependent on the expressing cells’ environment (Noel and Sounni 2013; Sternlicht and Werb 2001). An LEC may be able to produce more MT1-MMP or less TIMP2 when the existing stock of MT1-MMP in its environment is depleted. Furthermore, we are only modelling one LEC when each secondary sprout consists of multiple LECs. Our model certainly underestimates the collagenolytic effects of MMP2. We will perform a numerical experiment which replaces the activation mechanism of MMP2 with a constant production rate. This simplified model ignores proMMP2, TIMP2, MT1-MMP, and their complexes. Only the equations governing the interstitial flow, $\tilde{C}_{\text {M2}}$, $\tilde{C}_\mathrm{VC}$, $\tilde{C}_{\text {C1}}$, and $\tilde{C}_\mathrm{VC \cdot C1}$ remain. Furthermore, we will use $\tilde{R}^\mathrm{LEC}_{\text {M2}}=10$ and $\tilde{R}^\mathrm{IS}_{\text {M2}}=-\lambda _{15} \tilde{C}_{\text {M2}}$ in the modified model. In Fig. 7b, the maximum $\tilde{R}^\mathrm{LEC}_{\text {M2}}$ is around 0.2, so $\tilde{R}^\mathrm{LEC}_{\text {M2}}=10$ is a significant increase. The results of this numerical experiment are presented in Fig. 8. Similar to the primary simulation, MMP2 is almost homogeneously distributed. The flat MMP2 gradients explain why collagen I degradation does not vary significantly in space. On the other hand, the extent of degradation is significant, almost 20%.

To conclude, MMP2 diffusion in a zebrafish trunk can be considered to be in equilibrium on the time scale of lymphangiogenesis. When diffusion is the dominant mode of transport, MMP2 will permeate the entire embryo and degrade collagen I almost homogeneously. This is in agreement with the conceptual model proposed by Karagiannis and Popel (2006). The diffusible protease MMP2 remodels the embryo’s ECM, making it more conducive to cell migration; the LEC-bound protease MT1-MMP degrades the collagen I around the LECs, thereby triggering LEC migration. Another conclusion is about TIMP2. In the primary simulation, MMP2-TIMP2 binding does not affect the homogeneous distribution of MMP2, so TIMP2 diffusion is also in equilibrium . It only changes the baseline concentration of MMP2, not its spatial distribution. In this paper, MMP2-TIMP2 binding is only considered in the primary simulation.

### Convection and Asymmetry

The biologically relevant range of $C_{\text {C1}}$ is from $1.59\times 10^{-6}$ M to $3.50\times 10^{-4}$ M. Our choice of $C_\mathrm{C1,s}$ is $3.50\times 10^{-4}$ M, so the initial condition $\tilde{C}_{\text {C1}}=1$ falls at the upper end of the range.

We will carry out a numerical experiment where the initial $\tilde{C}_{\text {C1}}$ is uniformly reduced to 0.1. We will ignore collagen I degradation by MMP2 and MMP2-TIMP2 binding in the modified model, meaning it will only retain the interstitial flow Eqs. (27) and (48), as well as the governing equations for free VEGFC, collagen I, and VEGFC bound to collagen I. The two neglected phenomena can only reduce the baseline of $\tilde{C}_{\text {C1}}$ in a diffusion-dominant zebrafish embryo. Our manual reduction in the initial $\tilde{C}_{\text {C1}}$ has the same effect. A reduction in $\tilde{C}_{\text {C1}}$ will affect both the transport and kinetic terms in our model. On the other hand, there are ECM components other than collagen I in a real zebrafish embryo; changing the amount of collagen I present will affect the transport properties, but the ability of components like heparan sulphate to sequester VEGFC will not be affected. For this reason, we will modify the model to control the kinetic properties. Since we are lowering $\tilde{C}_{\text {C1}}$ uniformly by an order of magnitude, we will increase the binding terms of VEGFC and collagen I by an order of magnitude. It means the reaction terms will become $\tilde{R}^\mathrm{IS}_\mathrm{VC}=-10\lambda _{25}\tilde{C}_\mathrm{VC}\tilde{C}_{\text {C1}}+\lambda _{26}\tilde{C}_\mathrm{VC\cdot C1}-\lambda _{27}\tilde{C}_\mathrm{VC}$, $\tilde{R}^\mathrm{IS}_{\text {C1}}=-10\lambda _{29}\tilde{C}_\mathrm{VC}\tilde{C}_{\text {C1}}+\lambda _{30}\tilde{C}_\mathrm{VC\cdot C1}$, and $\tilde{R}^\mathrm{IS}_\mathrm{VC\cdot C1}=10\tilde{C}_\mathrm{VC}\tilde{C}_{\text {C1}}-\lambda _{32} \tilde{C}_\mathrm{VC\cdot C1}$.

Then, we will repeat the numerical experiment after switching off the interactions between VEGFC and collagen I. This means we will further simplify the model by eliminating the governing equations for collagen I and bound VEGFC. This modified model will only have the interstitial flow Eqs. (27) and (48) in addition to the reaction–diffusion–convection equation governing $\tilde{C}_\mathrm{VC}$. The reaction term $\tilde{R}^\mathrm{IS}_\mathrm{VC}$ is simplified to $-\lambda _{27}\tilde{C}_\mathrm{VC}$ and $\tilde{C}_{\text {C1}}$ is constant at 0.1.

**Fig. 9:**
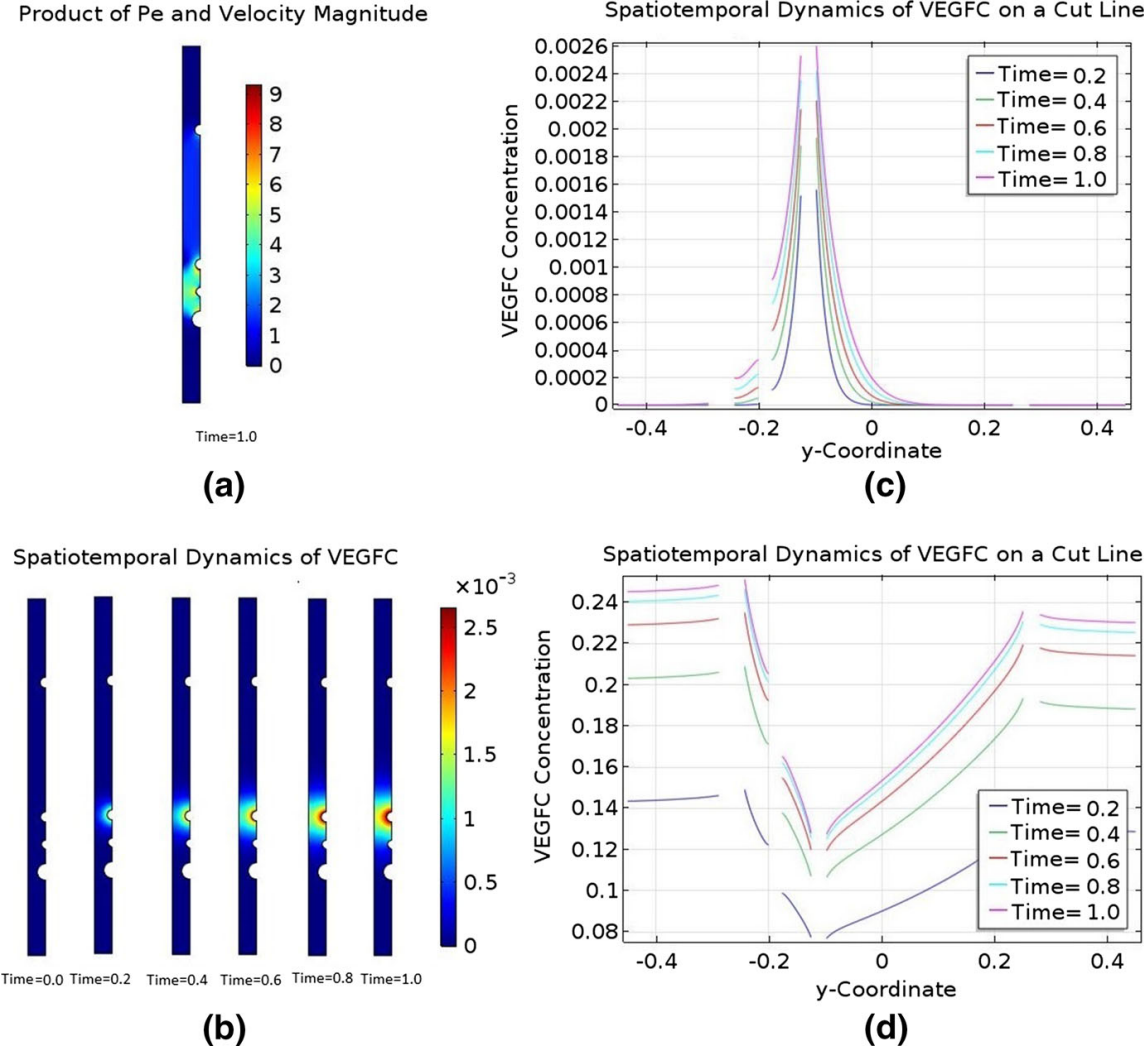
(Color figure online) Spatiotemporal dynamics of VEGFC in a marginally convection-dominant zebrafish embryo. The initial $\tilde{C}_{\text {C1}}$ is 0.1 and collagen I does not degrade. **a** That convection is marginally dominant in the central region of the embryo, but diffusion is still dominant in the periphery. **b** The concentration profiles of VEGFC at selected time points. VEGFC is sequestered by collagen I in this case. **c** Plots the data of (**b**) on the cut line running from $\tilde{y}=-0.45$ to $\tilde{y}=0.45$ at $\tilde{x}=0$. **d** The spatiotemporal dynamics of VEGFC on the same cut line when VEGFC does not interact with collagen I

**Fig. 10:**
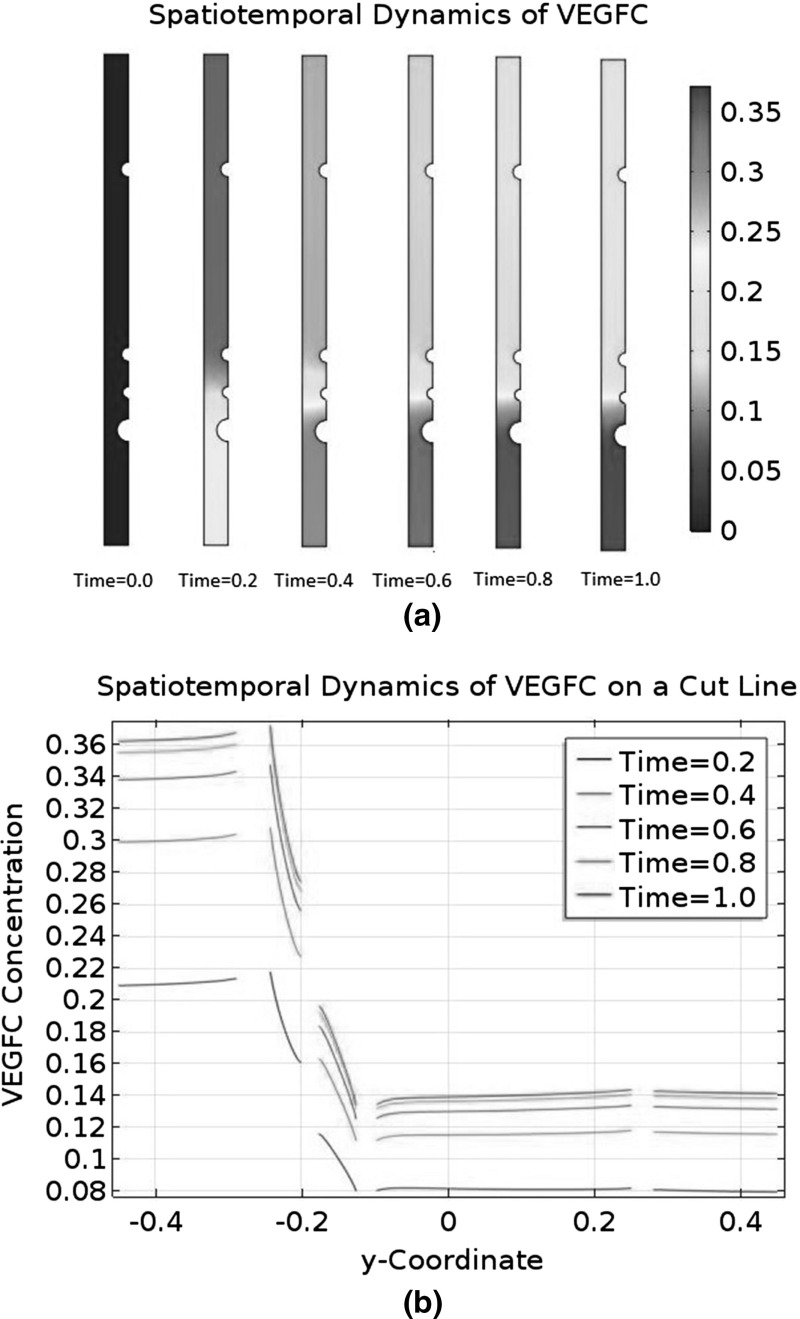
(Color figure online) Spatiotemporal dynamics of VEGFC in an asymmetric pressure field. The initial $\tilde{C}_{\text {C1}}$ is 0.1, and collagen I does not degrade. Convection is marginally dominant in the central region of the embryo, while diffusion dominates in the periphery. VEGFC does not interact with collagen I. **a** A collection of VEGFC concentration profiles at selected time points. **b** The spatiotemporal dynamics of VEGFC on the cut line running from $\tilde{y}=-0.45$ to $\tilde{y}=0.45$ at $\tilde{x}=0$

**Fig. 11:**
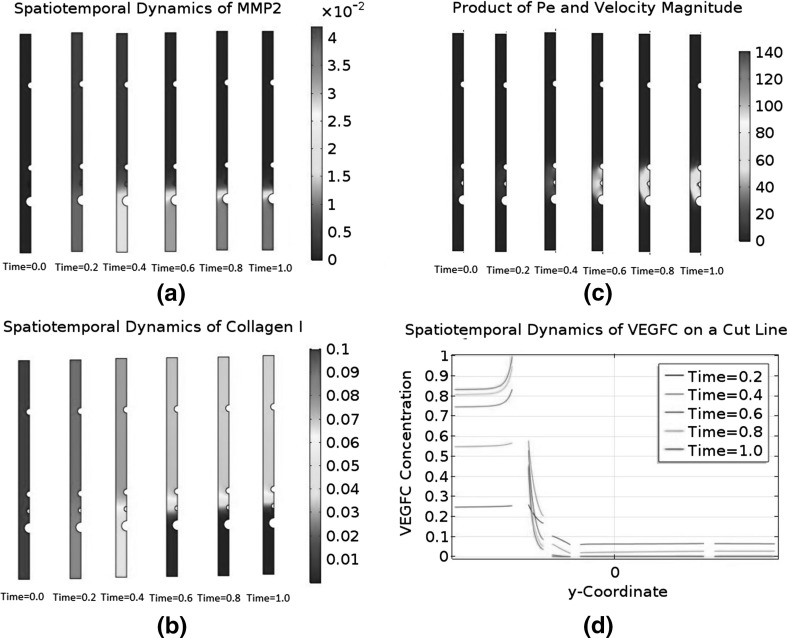
(Color figure online) Positive feedback loop between an asymmetric interstitial flow and the collagenolytic action of MMP2. Initially, $\tilde{C}_{\text {C1}}$ is 0.1 and convection is marginally dominant in the central region of the embryo, but diffusion dominates in the periphery. MMP2 is produced at a constant rate to degrade collagen I. VEGFC is not sequestered by collagen I. The pressure field is asymmetric. **a** The spatiotemporal dynamics of MMP2. **b** The spatiotemporal dynamics of collagen I. **c** That convection becomes increasingly dominant in the region ventral to the DA. **d** The spatiotemporal dynamics of VEGFC on the cut line running from $\tilde{y}=-0.45$ to $\tilde{y}=0.45$ at $\tilde{x}=0$

Figure 9 summarises the results of these two numerical experiments with the initial condition $\tilde{C}_{\text {C1}}=0.1$. From Fig. 9a, we can infer that convection marginally dominates diffusion in the centre of the embryo, but diffusion still dominates in the periphery. Figure 9b and c is very similar to their counterparts in Fig. 5 in terms of the baseline of $\tilde{C}_\mathrm{VC}$, its spatial variations, and the steepness of its gradients. When VEGFC does not bind to collagen I, its spatiotemporal dynamics in Fig. 9d are completely different. The interstitial flow pushes most of the embryo’s VEGFC to the periphery. The baseline of $\tilde{C}_\mathrm{VC}$ is two orders of magnitude higher than when VEGFC is sequestered by collagen I. However, the LEC is in a VEGFC gradient which only decreases twofold from the PCV to the DA. This shallow gradient is unsuitable for morphogenetic functions. Its directionality does not allow VEGFC to guide the migrating LEC to the horizontal myoseptum either.

We hypothesise that the pressure field is the key regulator of VEGFC’s spatiotemporal dynamics in the last numerical experiment. To test this hypothesis, we will repeat it with a steeper and asymmetric pressure field which is given by

56 $$\begin{aligned} \tilde{P^{s}}= & {} \frac{1}{\eta _{3}} \qquad \tilde{\varvec{x}} \in \partial \varOmega _\mathrm{DA}, \end{aligned}$$

57 $$\begin{aligned} \tilde{P^{s}}= & {} \frac{-0.5}{\eta _{3}} \qquad \tilde{\varvec{x}} \in \partial \varOmega _\mathrm{PCV},\; \text {and} \end{aligned}$$

58 $$\begin{aligned} \tilde{P^{s}}= & {} \frac{0.8}{\eta _{3}} \qquad \tilde{\varvec{x}} \in \partial \varOmega _\mathrm{DLAV}. \end{aligned}$$

Figure 10 illustrates the resulting dynamics of $\tilde{C}_\mathrm{VC}$. Steepening the pressure gradient from the DA to the PCV steepens the gradient of $\tilde{C}_\mathrm{VC}$ in that region too. It is now a threefold gradient, so VEGFC may act as a morphogen for the LEC. The pressure drop from the DA to the PCV is larger than that from the DA to the DLAV. The interstitial flow will thus be faster on the ventral side of the DA. VEGFC goes with the interstitial flow, forming an asymmetric concentration field. If VEGFC is a chemorepellent, it can guide the LEC down the gradient towards the horizontal myoseptum. Considering VEGFC promotes survival, proliferation, and migration in LECs, it is unlikely to be one. Nevertheless, the pressure field in the numerical experiment is arbitrary. Reversing its direction will allow VEGFC to chemoattract the LEC to its destination.

To conclude, there is a tension between convection and VEGFC sequestration by the ECM in an embryo. Even when convection is marginally dominant in the embryo, its interstitial flow cannot influence VEGFC when the latter is sequestered by the ECM. When VEGFC is influenced by the flow, the pressure field in the embryo is the key determinant of its spatiotemporal dynamics. Its steepness determines whether VEGFC can be a morphogen for the migrating LECs in the embryo; its directionality determines whether VEGFC can guide their migration by chemotaxis.

### Channelisation

Section 3.3 concludes that the spatial variations of MMP2 are small in a diffusion-dominant embryo. MMP2 will gradually render such an embryo convection-dominant without introducing significant spatial effects. The embryo described in Sect. 3.4 is convection-dominant through its initial $\tilde{C}_{\text {C1}}$. Section 3.4 concludes that asymmetric and steep concentration gradients are possible after the manual switch from diffusion to convection. The prerequisites are an asymmetric and steep pressure field and an ECM which does not sequester the diffusing species under consideration. However, we have not considered how MMP2 fits into the picture *after* it takes a diffusion-dominant embryo into its convection-dominant regime. It makes us wonder whether MMP2 will behave differently in a convection-dominant embryo. To investigate this possibility, we will perform a numerical experiment. Apart from the interstitial flow Eqs. (27) and (48), we will solve the governing equations for MMP2, VEGFC, and collagen I in this numerical experiment. MMP2 will be produced at a constant rate like the numerical experiment in Sect. 3.3. The reaction terms are $\tilde{R}^\mathrm{LEC}_{\text {M2}}=10$, $\tilde{R}^\mathrm{IS}_{\text {M2}}=-\lambda _{15} \tilde{C}_{\text {M2}}$, $\tilde{R}^\mathrm{IS}_\mathrm{VC}=-\lambda _{27}\tilde{C}_\mathrm{VC}$, and $\tilde{R}^\mathrm{IS}_{\text {C1}}=\frac{-\lambda _{28}\tilde{C}_{\text {M2}}\tilde{C}_{\text {C1}}}{K^\mathrm{M2,C1}_{M}+C_\mathrm{C1,s}\tilde{C}_{\text {C1}}}$. We will also use the asymmetric pressure field given by equations (56), (57), and (58). We will not model MMP2-TIMP2 binding because MMP2 and TIMP2 are produced at the same source and have similar diffusion coefficients, so this phenomenon will only affect the baseline concentration of MMP2, not its spatial distribution.

Figure 11 summarises the simulation results of this numerical experiment. There is a positive feedback loop between convection and ECM remodelling. Due to the asymmetric interstitial flow, MMP2 is concentrated at the ventral end of the embryo. This asymmetry in $\tilde{C}_{\text {M2}}$ results in preferential degradation of collagen I at the ventral end, resulting in a gradient of $\tilde{C}_{\text {C1}}$ which decreases ventrally from the DA. This gradient will strengthen the interstitial flow in that direction to complete the feedback loop. The impact of this loop on VEGFC is shown in Fig. 11d. Its concentration gradient steepens with time because it also follows the ever increasing interstitial flow. If VEGFC is a morphogen and chemotactic factor for LECs, this positive feedback loop enhances these functions.

**Fig. 12:**
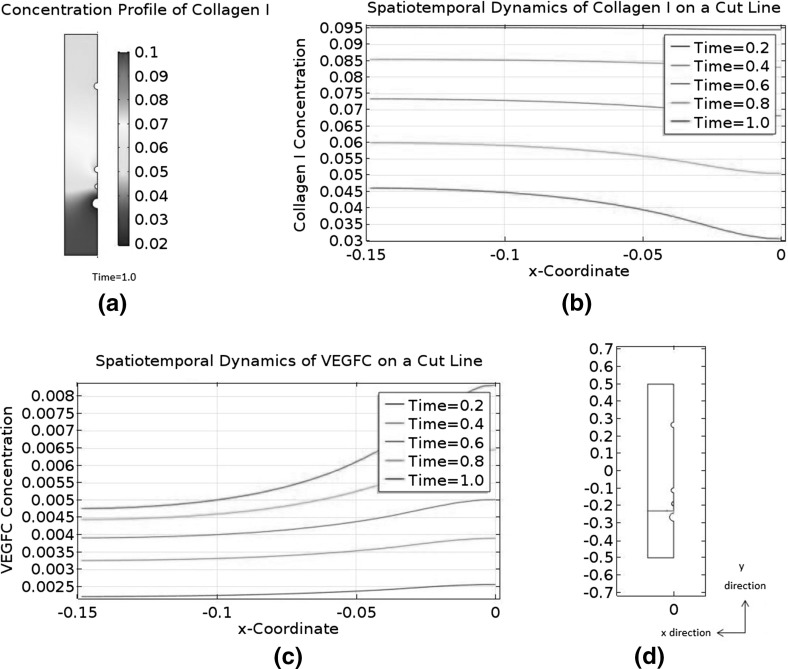
(Color figure online) Channels form in collagen I due to an asymmetric interstitial flow and the collagenolytic action of MMP2. Initially, $\tilde{C}_{\text {C1}}$ is 0.1 and convection is marginally dominant in the central region of the embryo, but diffusion dominates in the periphery. MMP2 is produced at a constant rate to degrade collagen I. VEGFC is not sequestered by collagen I. The pressure field is asymmetric. **a** The concentration profile of collagen I at $\tilde{t}=1$. **b** The spatiotemporal dynamics of collagen I on the cut line running from $\tilde{x}=-0.15$ (outer boundary) to $\tilde{x}=0$ (line of symmetry) at $\tilde{y}=-0.23$. This cut line is between the posterior cardinal vein and the lymphatic endothelial cell. **c** The spatiotemporal dynamics of VEGFC on the same cut line. **d** The aforementioned cut line from $\tilde{x}=-0.15$ to $\tilde{x}=0$ at $\tilde{y}=-0.23$

Our idealised zebrafish embryo is too thin for any spatial effects to show up in the x-direction. Considering our dimensions are only estimates, we will repeat the last numerical experiment with a triply widened embryo. The modified geometry will go from $\tilde{x}=-0.15$ (outer boundary) to $\tilde{x}=0$ (line of symmetry). The simulation results are in Fig. 12. Figure 12a, between the DA and the PCV, clearly shows some variations in $\tilde{C}_{\text {C1}}$ in the x-direction. Therefore, we will focus on the cut line from $\tilde{x}=-0.15$ to $\tilde{x}=0$ at $\tilde{y}=-0.23$, a point between the LEC and the PCV. Figure 12b illustrates that a channel with low $\tilde{C}_{\text {C1}}$ forms gradually in the plane where the LEC and the blood vessels are located. Figure 12c indicates that VEGFC accumulates in this channel. We know that VEGFC is a growth factor for LECs, while collagen I is a physical barrier to their migration. Therefore, they are likely to stay in the channel. Channelisation ensures the LECs will migrate in the y-direction and the lymphatic vessels they form will lie in the same plane as the blood vessels.

To conclude, an asymmetric interstitial flow and the collagenolytic action of MMP2 form a positive feedback loop in a zebrafish embryo. The outcome is a channel of low $\tilde{C}_{\text {C1}}$ and high $\tilde{C}_\mathrm{VC}$ near the blood vessels’ plane. The channel can keep the embryo’s LECs migrating on a ventral–dorsal path, so the resulting lymphatic vessels will stay close to the blood vessels.

### Concentration Gradients Vanish When Collagen I is Insufficient

Finally, we will consider the case where the initial $\tilde{C}_{\text {C1}}$ is 0.01. Since there is so little collagen I to begin with, we will not consider its degradation by MMP2 and without MMP2, and we will not consider TIMP2 either. Therefore, the modified model will only include the interstitial flow Eqs. (27) and (48) and the governing equations for VEGFC, collagen I, and VEGFC bound to collagen I. To control for the kinetic properties in our model, we will increase the binding terms of VEGFC and collagen I by two orders of magnitude. Therefore, the reaction terms are $\tilde{R}^\mathrm{IS}_\mathrm{VC}=-100\lambda _{25}\tilde{C}_\mathrm{VC}\tilde{C}_{\text {C1}}+\lambda _{26}\tilde{C}_\mathrm{VC\cdot C1}-\lambda _{27}\tilde{C}_\mathrm{VC}$, $\tilde{R}^\mathrm{IS}_{\text {C1}}=-100\lambda _{29}\tilde{C}_\mathrm{VC}\tilde{C}_{\text {C1}}+\lambda _{30}\tilde{C}_\mathrm{VC\cdot C1}$, and $\tilde{R}^\mathrm{IS}_\mathrm{VC\cdot C1}=100\tilde{C}_\mathrm{VC}\tilde{C}_{\text {C1}}-\lambda _{32} \tilde{C}_\mathrm{VC\cdot C1}$. We can further simplify the effective diffusivity of VEGFC too. The term inside the brackets in $\lambda _\mathrm{1,VC}\left( 1-\lambda _\mathrm{2,VC}\sqrt{\lambda _{3}\tilde{C}_{\text {C1}}}\right)$ is between 0.94 and 1 now. Approximating it as unity, we will retain $\lambda _\mathrm{1,VC}$ only.

**Fig. 13:**
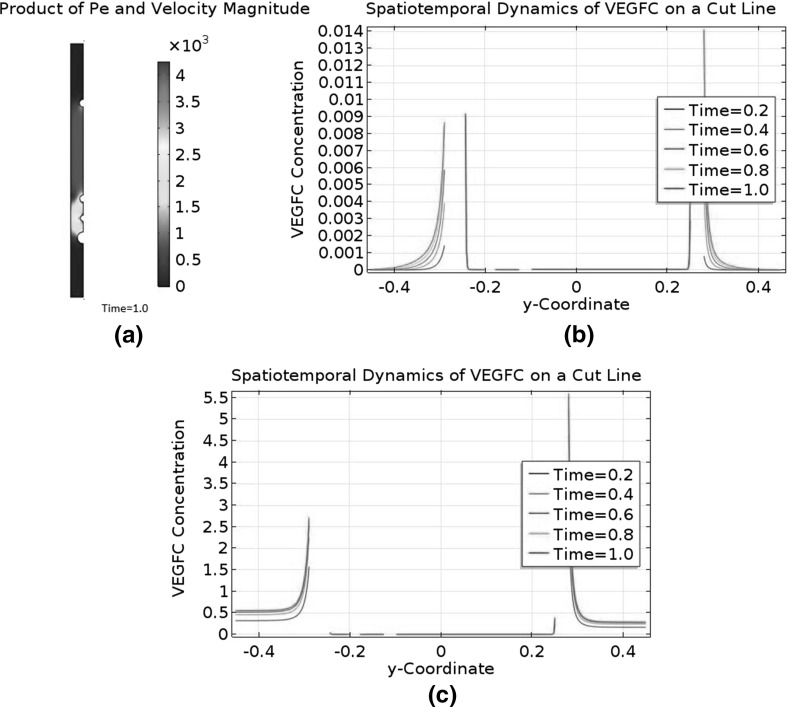
(Color figure online) Concentration gradients cannot form with insufficient collagen I. In these two numerical experiments, the initial $\tilde{C}_{\text {C1}}$ is 0.01 and there is no collagen I degradation. **a** The central region of the embryo is overwhelmingly dominated by convection although diffusion dominates in the periphery. **b** The spatiotemporal dynamics of VEGFC on the cut line running from $\tilde{y}=-0.45$ to $\tilde{y}=0.45$ at $\tilde{x}=0$. In this numerical experiment, VEGFC is sequestered by collagen I. **c** The same dynamics as (**b**) when VEGFC does not interact with collagen I

Figure 13a and b summarises the simulation results of this numerical experiment. The $|\tilde{\varvec{u}}|Pe$ profile shows that convection is orders of magnitude more important than diffusion in the central region of the embryo. As a result, the interstitial flow flushes all the VEGFC in the embryo to the periphery where it forms boundary layers. These gradients cannot influence the LEC in the central region. We will repeat the numerical experiment by neglecting the interactions between VEGFC and collagen I. This means only the interstitial flow equations and the governing equation for VEGFC will be solved, $\tilde{R}^\mathrm{IS}_\mathrm{VC}=-\lambda _{27}\tilde{C}_\mathrm{VC}$, and $\tilde{C}_{\text {C1}}$ is constant at 0.01. Figure 13c shows the resulting spatiotemporal dynamics of VEGFC. The central regions of Fig. 13b and c are the same, proving that sequestration by collagen I cannot protect VEGFC from such a powerful interstitial flow.

To conclude, when collagen I is insufficient, the interstitial flow in a zebrafish embryo will clear its central region of VEGFC. As a result, VEGFC cannot form a suitable concentration gradient to guide the embryo’s LECs as a morphogen or a chemotactic factor. When the interstitial flow is strong enough, sequestration by collagen I cannot protect VEGFC from going with the flow. However, our pressure field is only an estimate. The ECM contains components other than collagen I too. Our hydraulic conductivity and interstitial flow rate in this subsection are probably overestimates and not representative of zebrafish embryos.

## Discussion

Our study proposes a model of the spatiotemporal dynamics of VEGFC in the trunk of a zebrafish embryo. The model parameters come from different studies. Many of them do not have in vivo sources, and many more do not pertain to zebrafish embryos. Some of them are simply unavailable, such as our use of a reported VEGF production rate as a surrogate for the required VEGFC production rate. To complicate matters, the conditions in an embryo are dynamic, so the parameters should not be static as they are in our model. Therefore, we cannot use the model to simulate what actually happens in a zebrafish embryo. Nonetheless, our simulations are scenarios within the realms of possibility. There are three scenarios in which VEGFC can be a morphogen or chemotactic factor for the LECs migrating from the PCV to the horizontal myoseptum of a zebrafish embryo.

### Scenario 1

This scenario is a diffusion-dominant embryo where VEGFC is sequestered by the ECM. Under these circumstances, VEGFC will have a concentration profile which peaks at and decreases symmetrically from its source, the DA. The gradient between the PCV and the DA is perfect for VEGFC to act as a morphogen for the migrating LECs. However, the symmetry of the profile makes VEGFC an unlikely candidate for their chemotactic factor.

In general, the combination of diffusion and sequestration by an ECM creates short-range, steep, and symmetric gradients of mobile species.

### Scenario 2

Scenario 2 is an embryo marginally dominated by convection. When VEGFC binds to the embryo’s ECM, it is protected from the interstitial flow therein. VEGFC will behave like it does in scenario 1.

### Scenario 3

Scenario 3 is also an embryo marginally dominated by convection, but VEGFC does not bind to the ECM. In this case, the pressure field is the key factor regulating the behaviour of VEGFC. A sufficiently steep pressure field will establish a VEGFC concentration gradient appropriate for morphogenetic functions. An asymmetric pressure field will give a directionality to the gradient, thus allowing VEGFC to guide cell migration by chemotaxis.

In general, a steep and asymmetric pressure field will create steep, asymmetric, and embryo-wide concentration gradients of mobile species. The prerequisites are convection being marginally dominant and a nonbinding ECM.

### Collagen I Degradation is a Control Mechanism

Collagen I concentration is the key variable that determines the dominant mode of transport, hence the scenario which applies to an embryo. Collagen I degradation by MMP2 is therefore an important means to control the behaviour of VEGFC. There are two ways MMP2 can exert its influence.

First, it allows an embryo to switch back and forth between the three scenarios. When diffusion is dominant, the distribution of MMP2 in the embryo is almost homogeneous. Collagen I will degrade almost uniformly until convection takes over. Conversely, although our model cannot demonstrate this feature, the embryo can lower MMP2 production and increase collagen I production, thus tilting the balance in diffusion’s favour. This control mechanism is useful for patterning the embryo. The embryo can start in scenario 3 where embryo-wide morphogen gradients divide it into segments of different cell types. Then, it can switch to scenario 1 where local gradients will fine-tune the development of each segment.

Second, MMP2 and an asymmetric interstitial flow can form a positive feedback mechanism in scenario 3. The flow goes asymmetrically on the ventral–dorsal axis. It will concentrate MMP2 in one direction on this axis. Collagen I will as a result degrade preferentially in this direction, further skewing the interstitial flow. VEGFC will also go with the flow, thus steepening its own concentration gradient. The increased steepness makes VEGFC a more effective morphogen, while the increased asymmetry makes it a more effective chemotactic factor. Because collagen I degrades preferentially on one axis, a channel of abundant VEGFC and scarce collagen I will form along this axis. Because VEGFC is a growth factor and collagen I is a barrier to cell migration, the embryo’s LECs will migrate in the channel. Channelisation therefore ensures LEC migration occurs on the ventral–dorsal axis.

Finally, when collagen I is insufficient, the embryo will be overwhelmingly dominated by convection. The interstitial flow will be so strong that no gradients of mobile species can form in the embryo. This scenario is unlikely to occur in an embryo, however.

### Future Work

The developmental processes of living organisms are shaped by evolution. The fact that VEGFC is distributed like a morphogen in multiple scenarios suggests that it is actually one. On the other hand, even if VEGFC is a chemoattractant or chemorepellent, it can only perform its function in an asymmetric pressure field in scenario 3. A different molecular species is probably required to guide the PCV-derived LECs to the horizontal myoseptum in a zebrafish embryo. However, to validate our predictions, we need to comprehend the intracellular responses of an LEC to a concentration gradient of VEGFC.

Even if a species other than VEGFC induces the PCV-derived LECs to differentiate, it probably forms an appropriate concentration profile in one of the scenarios this study proposes. The proposed control mechanisms probably regulate its spatiotemporal dynamics too. More generally, these scenarios and mechanisms are frameworks within which embryonic development and tissue regeneration can be understood.

The mathematical model per se is a valuable tool for mathematical and theoretical biologists. To the best of our knowledge, it is the first mathematical model that considers an interstitial flow, a remodelling ECM, and the ECM’s ability to sequester a mobile species together. Its solution costs very little computationally, so it can be extended to more complex geometries, larger domains, and higher dimensions. By changing the model geometry, its biochemical reaction network, and the model parameters, one can simulate the dynamics of a diverse range of biological phenomena.

## References

[CR1] Abramowitz M, Stegun IA (1964). Handbook of mathematical functions with formulas, graphs, and mathematical tables, ninth dover printing.

[CR2] Berk D, Yuan F, Leunig M, Jain R (1993). Fluorescence photobleaching with spatial fourier analysis: measurement of diffusion in light-scattering media. Biophys J.

[CR3] Bruyère F, Melen-Lamalle L, Blacher S, Roland G, Thiry M, Moons L, Frankenne F, Carmeliet P, Alitalo K, Libert C (2008). Modeling lymphangiogenesis in a three-dimensional culture system. Nat Methods.

[CR4] Bussmann J, Bos FL, Urasaki A, Kawakami K, Duckers HJ, Schulte-Merker S (2010). Arteries provide essential guidance cues for lymphatic endothelial cells in the zebrafish trunk. Development.

[CR5] Cha YR, Fujita M, Butler M, Isogai S, Kochhan E, Siekmann AF, Weinstein BM (2012). Chemokine signaling directs trunk lymphatic network formation along the preexisting blood vasculature. Dev Cell.

[CR6] Chen H, Griffin C, Xia L, Srinivasan RS (2014). Molecular and cellular mechanisms of lymphatic vascular maturation. Microvasc Res.

[CR7] Coffindaffer-Wilson M, Craig MP, Hove JR (2011). Normal interstitial flow is critical for developmental lymphangiogenesis in the zebrafish. Lymphat Res Biol.

[CR8] Coffindaffer-Wilson M, Criag M, Hove J (2011). Determination of lymphatic vascular identity and developmental timecourse in zebrafish (*Danio rerio*). Lymphology.

[CR9] Deng Y, Atri D, Eichmann A, Simons M (2013). Endothelial erk signaling controls lymphatic fate specification. J Clin Invest.

[CR10] Detry B, Erpicum C, Paupert J, Blacher S, Maillard C, Bruyère F, Pendeville H, Remacle T, Lambert V, Balsat C (2012). Matrix metalloproteinase-2 governs lymphatic vessel formation as an interstitial collagenase. Blood.

[CR11] Edds MV (1958). Development of collagen in the frog embryo. Proc Natl Acad Sci USA.

[CR12] Einstein A (1905). Über die von der molekularkinetischen theorie der wärme geforderte bewegung von in ruhenden flüssigkeiten suspendierten teilchen. Ann Phys.

[CR13] Frcitas RA (1998). Exploratory design in medical nanotechnology: a mechanical artificial red cell. Artif Cells Blood Substit Biotechnol.

[CR14] Gore AV, Monzo K, Cha YR, Pan W, Weinstein BM (2012). Vascular development in the zebrafish. Cold Spring Harb Perspect Med.

[CR15] Gurdon J, Bourillot PY (2001). Morphogen gradient interpretation. Nature.

[CR16] Gutfreund H (1993) Proteins: structures and molecular properties

[CR17] Hashambhoy YL, Chappell JC, Peirce SM, Bautch VL, Mac Gabhann F (2011) Computational modeling of interacting vegf and soluble vegf receptor concentration gradients. Front Physiol 62:1–12 10.3389/fphys.2011.00062 PMC3185289 22007175

[CR18] Helm CLE, Zisch A, Swartz MA (2007). Engineered blood and lymphatic capillaries in 3-d vegf-fibrin-collagen matrices with interstitial flow. Biotechnol Bioeng.

[CR19] Hermans K, Claes F, Vandevelde W, Zheng W, Geudens I, Orsenigo F, De Smet F, Gjini E, Anthonis K, Ren B (2010). Role of synectin in lymphatic development in zebrafish and frogs. Blood.

[CR20] Hogan BM, Bos FL, Bussmann J, Witte M, Chi NC, Duckers HJ, Schulte-Merker S (2009). Ccbe1 is required for embryonic lymphangiogenesis and venous sprouting. Nat Genet.

[CR21] Hu N, Sedmera D, Yost HJ, Clark EB (2000). Structure and function of the developing zebrafish heart. Anat Rec.

[CR22] Iida A, Sakaguchi K, Sato K, Sakurai H, Nishimura D, Iwaki A, Takeuchi M, Kobayashi M, Misaki K, Yonemura S (2010). Metalloprotease-dependent onset of blood circulation in zebrafish. Curr Biol.

[CR23] Isogai S, Lawson ND, Torrealday S, Horiguchi M, Weinstein BM (2003). Angiogenic network formation in the developing vertebrate trunk. Development.

[CR24] Jain RK (1987). Transport of molecules across tumor vasculature. Cancer Metastasis Rev.

[CR25] Jeltsch M, Jha SK, Tvorogov D, Anisimov A, Leppänen VM, Holopainen T, Kivelä R, Ortega S, Kärpanen T, Alitalo K (2014). Ccbe1 enhances lymphangiogenesis via a disintegrin and metalloprotease with thrombospondin motifs-3-mediated vascular endothelial growth factor-c activation. Circulation.

[CR26] Ji RC (2006). Lymphatic endothelial cells, lymphangiogenesis, and extracellular matrix. Lymphat Res Biol.

[CR27] Joukov V, Pajusola K, Kaipainen A, Chilov D, Lahtinen I, Kukk E, Saksela O, Kalkkinen N, Alitalo K (1996). A novel vascular endothelial growth factor, vegf-c, is a ligand for the flt4 (vegfr-3) and kdr (vegfr-2) receptor tyrosine kinases. EMBO J.

[CR28] Joukov V, Sorsa T, Kumar V, Jeltsch M, Claesson-Welsh L, Cao Y, Saksela O, Kalkkinen N, Alitalo K (1997). Proteolytic processing regulates receptor specificity and activity of vegf-c. EMBO J.

[CR29] Karagiannis ED, Popel AS (2004). A theoretical model of type i collagen proteolysis by matrix metalloproteinase (mmp) 2 and membrane type 1 mmp in the presence of tissue inhibitor of metalloproteinase 2. J Biol Chem.

[CR30] Karagiannis ED, Popel AS (2006). Distinct modes of collagen type i proteolysis by matrix metalloproteinase (mmp) 2 and membrane type i mmp during the migration of a tip endothelial cell: insights from a computational model. J Theor Biol.

[CR31] Köhn-Luque A, de Back W, Yamaguchi Y, Yoshimura K, Herrero M, Miura T (2013). Dynamics of vegf matrix-retention in vascular network patterning. Phys Biol.

[CR32] Koltowska K, Betterman KL, Harvey NL, Hogan BM (2013). Getting out and about: the emergence and morphogenesis of the vertebrate lymphatic vasculature. Development.

[CR33] Levick J (1987). Flow through interstitium and other fibrous matrices. Q J Exp Physiol.

[CR34] Louveau A, Smirnov I, Keyes TJ, Eccles JD, Rouhani SJ, Peske JD, Derecki NC, Castle D, Mandell JW, Lee KS, et al (2015) Structural and functional features of central nervous system lymphatic vessels. Nature 523:337–341 10.1038/nature14432 PMC4506234 26030524

[CR35] Lutter S, Makinen T (2014) Regulation of lymphatic vasculature by extracellular matrix. In: Kiefer F, Schulte-Merker S (eds) Developmental aspects of the lymphatic vascular system, Springer, Berlin, pp 55–65 10.1007/978-3-7091-1646-3_5 24276886

[CR36] Mäkinen T, Veikkola T, Mustjoki S, Karpanen T, Catimel B, Nice EC, Wise L, Mercer A, Kowalski H, Kerjaschki D (2001). Isolated lymphatic endothelial cells transduce growth, survival and migratory signals via the vegf-c/d receptor vegfr-3. EMBO J.

[CR37] Margaris K, Black RA (2012). Modelling the lymphatic system: challenges and opportunities. J R Soc Interf.

[CR38] McGee SP, Cooper EM, Stapleton HM, Volz DC (2012). Early zebrafish embryogenesis is susceptible to developmental tdcpp exposure. Environ Health Perspect.

[CR39] Ng CP, Helm CLE, Swartz MA (2004). Interstitial flow differentially stimulates blood and lymphatic endothelial cell morphogenesis in vitro. Microvasc Res.

[CR40] Nicenboim J, Malkinson G, Lupo T, Asaf L, Sela Y, Mayseless O, Gibbs-Bar L, Senderovich N, Hashimshony T, Shin M, et al (2015) Lymphatic vessels arise from specialized angioblasts within a venous niche. Nature 522:56–61 10.1038/nature14425 25992545

[CR41] Noel A, Sounni NE (2013) MMP-mediated collagen remodeling and vessel functions. In: Brix K, Stöcker W (eds) Proteases: structure and function, Springer, Berlin, pp 471–489

[CR42] Ogston AG, Preston BN, Wells JD (1973). On the transport of compact particles through solutions of chain-polymers. Proc R Soc Lond A Math Phys Eng Sci.

[CR43] Paupert J, Sounni NE, Noël A (2011). Lymphangiogenesis in post-natal tissue remodeling: lymphatic endothelial cell connection with its environment. Mol Aspects Med.

[CR44] Prockop DJ, Kivirikko KI (1995). Collagens: molecular biology, diseases, and potentials for therapy. Annu Rev Biochem.

[CR45] Roose T, Tabor G (2013) Multiscale modelling of lymphatic drainage. In: Gefen A (ed) Multiscale computer modeling in biomechanics and biomedical engineering, Springer, Heidelberg, pp 149–176

[CR46] Sabin FR (1902). On the origin of the lymphatic system from the veins and the development of the lymph hearts and thoracic duct in the pig. Am J Anat.

[CR47] Schulte-Merker S, Sabine A, Petrova TV (2011). Lymphatic vascular morphogenesis in development, physiology, and disease. J Cell Biol.

[CR48] Shi ZD, Tarbell JM (2011). Fluid flow mechanotransduction in vascular smooth muscle cells and fibroblasts. Ann Biomed Eng.

[CR49] Siegfried G, Basak A, Cromlish JA, Benjannet S, Marcinkiewicz J, Chrétien M, Seidah NG, Khatib AM (2003). The secretory proprotein convertases furin, pc5, and pc7 activate vegf-c to induce tumorigenesis. J Clin Investig.

[CR50] Sternlicht MD, Werb Z (2001). How matrix metalloproteinases regulate cell behavior. Annu Rev Cell Dev Biol.

[CR51] Swartz MA, Fleury ME (2007). Interstitial flow and its effects in soft tissues. Annu Rev Biomed Eng.

[CR52] Toth M, Hernandez-Barrantes S, Osenkowski P, Bernardo MM, Gervasi DC, Shimura Y, Meroueh O, Kotra LP, Gálvez BG, Arroyo AG (2002). Complex pattern of membrane type 1 matrix metalloproteinase shedding regulation by autocatalytic cell surface inactivation of active enzyme. J Biol Chem.

[CR53] van Impel A, Schulte-Merker S (2014) A fisheye view on lymphangiogenesis. In: Kiefer F, Schulte-Merker S (eds) Developmental aspects of the lymphatic vascular system, Springer, Berlin, pp 153–165 10.1007/978-3-7091-1646-3_12 24276893

[CR54] Vempati P, Mac Gabhann F, Popel AS (2010). Quantifying the proteolytic release of extracellular matrix-sequestered vegf with a computational model. PLoS ONE.

[CR55] Wiig H, Keskin D, Kalluri R (2010). Interaction between the extracellular matrix and lymphatics: consequences for lymphangiogenesis and lymphatic function. Matrix Biol.

[CR56] Yaniv K, Isogai S, Castranova D, Dye L, Hitomi J, Weinstein BM (2006). Live imaging of lymphatic development in the zebrafish. Nat Med.

